# The archaeal glutamate transporter homologue Glt_Ph_ shows heterogeneous substrate binding

**DOI:** 10.1085/jgp.202213131

**Published:** 2022-04-22

**Authors:** Krishna D. Reddy, Didar Ciftci, Amanda J. Scopelliti, Olga Boudker

**Affiliations:** 1 Department of Physiology and Biophysics, Weill Cornell Medicine, New York, NY; 2 Tri-Institutional Training Program in Chemical Biology, New York, NY; 3 Howard Hughes Medical Institute, Weill Cornell Medicine, New York, NY

## Abstract

Integral membrane glutamate transporters couple the concentrative substrate transport to ion gradients. There is a wealth of structural and mechanistic information about this protein family. Recent studies of an archaeal homologue, Glt_Ph_, revealed transport rate heterogeneity, which is inconsistent with simple kinetic models; however, its structural and mechanistic determinants remain undefined. Here, we demonstrate that in a mutant Glt_Ph_, which exclusively populates the outward-facing state, at least two substates coexist in slow equilibrium, binding the substrate with different apparent affinities. Wild type Glt_Ph_ shows similar binding properties, and modulation of the substate equilibrium correlates with transport rates. The low-affinity substate of the mutant is transient following substrate binding. Consistently, cryo-EM on samples frozen within seconds after substrate addition reveals the presence of structural classes with perturbed helical packing of the extracellular half of the transport domain in regions adjacent to the binding site. By contrast, an equilibrated structure does not show such classes. The structure at 2.2-Å resolution details a pattern of waters in the intracellular half of the domain and resolves classes with subtle differences in the substrate-binding site. We hypothesize that the rigid cytoplasmic half of the domain mediates substrate and ion recognition and coupling, whereas the extracellular labile half sets the affinity and dynamic properties.

## Teaser

Glt_Ph_ is a model glutamate transporter with a kinetic heterogeneity of transport. Reddy et al. show that substrate binding is heterogeneous and suggest that subtle structural differences distinguish transporter subpopulations with different dynamic properties.

## Introduction

Membrane glutamate transporters pump substrates against their concentration gradients, serving critical biological functions across all kingdoms of life. In mammals, excitatory amino acid transporters recycle glutamate from the synaptic cleft into the glia (Freidman et al., 2020). In prokaryotes, orthologs take up nutrients, including glutamate, aspartate, neutral amino acids, or dicarboxylic acids (Kim et al., 2002; Burguiere et al., 2004; Youn et al., 2009). Transporters utilize energy from downhill ionic electrochemical gradients to carry concentrative substrate uptake. Excitatory amino acid transporters rely on inward Na^+^ and proton gradients and an outward K^+^ gradient (Zerangue and Kavanaugh, 1996). Prokaryotes couple transport to either proton or Na^+^ gradients (Tolner et al., 1995; Ryan et al., 2009).

These transporters are homotrimers with each protomer composed of a rigid scaffold trimerization domain and a mobile transport domain containing the ligand-binding sites. Protomers functions independently (Erkens et al., 2013; Ruan et al., 2017; Riederer et al., 2018; Georgieva et al., 2013; Koch et al., 2007; Grewer et al., 2005; Koch and Larsson, 2005). Transport domains translocate ligands across membranes by moving ∼15 Å from the outward-facing state (OFS) to the inward-facing state (IFS), in an elevator motion (Reyes et al, 2009; Garaeva et al, 2019; Arkhipova et al, 2020; Qiu et al, 2021). Studies in archaeal Na^+^-coupled transporters Glt_Ph_ and Glt_Tk_ led to a simple kinetic model of transport (Boudker et al., 2007; Reyes et al., 2013; Verdon et al., 2014; Oh and Boudker, 2018; Guskov et al., 2016; Riederer and Valiyaveetil, 2019; Wang and Boudker, 2020; Arkhipova et al., 2020; Alleva et al., 2020). Briefly, in the OFS, ion binding to Na1 and Na3 sites reveals the substrate and an additional sodium (Na2)-binding site through an opening of helical hairpin 2 (HP2), also preventing the translocation of Na^+^-only bound transport domain. Subsequent binding of the substrate and Na2 closes HP2, allowing translocation to the IFS and ligand release into the cytoplasm.

Recently, high-speed atomic force microscopy, single-molecule FRET (smFRET) TIRF microscopy, and ^19^F-NMR revealed a more complex picture of Glt_Ph_ transport and dynamics (Huang et al., 2020; Ciftci et al., 2020; Akyuz et al., 2015; Matin et al., 2020; Erkens et al., 2013; Akyuz et al., 2013; Huysmans et al., 2021; Ciftci et al., 2021). These studies established the existence of additional conformational substates in OFS and IFS, of which some translocate and transport at different rates. Although it is expected that cryo-EM studies would resolve these proposed conformational substates from heterogeneous datasets, this so far does not appear to be the case (Wang and Boudker, 2020; Arkhipova et al., 2020). Therefore, the structural and mechanistic determinants of kinetic heterogeneity remain unclear.

Using a Glt_Ph_ mutant that exclusively occupies the OFS, we show that substrate binding in the OFS is heterogeneous, consistent with at least two substates with different affinities. Salt composition and temperature modulate the substate populations, suggesting that they are in equilibrium. However, the conformational exchange must be slow, in the order of at least tens of seconds, for the substates to manifest in binding isotherms. Notably, a similar conformational equilibrium also exists in WT protein and potentially contributes to heterogeneous transport kinetics. The substrate-bound high-affinity state of the mutant transporter is expected to predominate after equilibration. Thus, to gain insights into the structure of the transient low-affinity substate, we conducted an extensive analysis of the cryo-EM imaging data collected on the transporter frozen within seconds after substrate addition. The identified structural classes reveal subtle differences. Specifically, we observed a subset of structural classes with differently packed helices in the extracellular half of the transport domain adjacent to the binding site, suggesting that the region is labile. We hypothesize that the ensemble of transient, more dynamic, less uniquely packed conformations comprises the low-affinity substate. Images of an equilibrated protein produced reconstructions at a uniquely high 2.2 Å resolution, revealing a complement of structured waters in the cytoplasmic side of the transport domain that may contribute to its conformational rigidity. We did not observe a conformational heterogeneity of the extracellular half of the transport domain in these data, which we attribute to the relaxation of the protein to the higher affinity state. Classifications instead revealed subtle differences in the substrate-binding site and the global orientations of the transport domains, which could also contribute to kinetic heterogeneity. Our results provide a framework in which modal kinetic behavior demonstrated by Glt_Ph_ may be a result of subtle but long-lived structural differences.

## Materials and methods

### DNA manipulations and protein preparation

Mutations were introduced to the previously described Glt_Ph_ CAT7 construct (Yernool et al., 2004) using PfuUltra II, and sequences were verified using Sanger sequencing (Macrogen). Proteins were expressed as C-terminal (His)_8_ fusions, separated by a thrombin cleavage site. Proteins were purified as previously described (Yernool et al., 2004). Briefly, crude membranes of DH10B *Escherichia coli* cells overexpressing Glt_Ph_ were solubilized for 2 h in 20 mM HEPES/NaOH, pH 7.4, 200 mM NaCl, 5 mM monopotassium L-aspartate (L-Asp), and 40 mM *n*-dodecyl-β-D-maltopyranoside (DDM; Anatrace). After solubilization, the DDM was diluted to ∼8–10 mM, and after high-speed ultracentrifugation (40,000 rpm, Ti45 rotor), the supernatant was applied to pre-equilibrated Ni-NTA affinity resin (Qiagen) for 1 h. The resin was washed with seven column volumes of 20 mM HEPES/NaOH, pH 7.4, 200 mM NaCl, 5 mM L-Asp, and 40 mM imidazole. Subsequently, the resin was eluted with the same buffer with increased imidazole (250 mM). The (His)_8_ tag was cleaved by thrombin digestion overnight at 4°C, and the proteins were further purified by size-exclusion chromatography (SEC) in the appropriate buffer for subsequent experiments. The concentration of Glt_Ph_ protomers was determined in a UV cuvette with a 10-mm pathlength (Starna Cells), using protein diluted 1:40, and an experimentally determined extinction coefficient of 57,400 M^−1^ cm^−1^ (Reyes et al., 2013).

### Reconstitution and uptake assays

Liposomes able to maintain proton gradients were prepared using a 3:1 mixture of POPE and POPG (1-palmitoyl-2-oleoyl-sn-glycero-3-phosphoethanolamine and 1-palmitoyl-2-oleoyl-sn-glycero-3-phospho-[1′-rac-glycerol]). Lipids were dried on the rotary evaporator for 2 h and under vacuum overnight. The resulting lipid film was hydrated by 10 freeze–thaw cycles at a concentration of 5 mg/ml in 50 mM potassium phosphate buffer and 100 mM potassium acetate, pH 7. The suspensions were extruded using a Mini-Extruder (Avanti) through 400-nm membranes (Whatman) 10 times to form unilamellar liposomes, and Triton X-100 was added to liposomes at a ratio of 1:2 (wt/wt).

P-Glt_Ph_ for reconstitution was affinity purified, thrombin cleaved, and purified by SEC in 20 mM HEPES/Tris, pH 7.4, 200 mM NaCl, 1 mM L-Asp, and 7 mM DDM. Purified protein was added to destabilized liposomes at a ratio of 1:1,000 (wt/wt) and incubated for 30 min at 23°C. Detergent was removed with four rounds of incubation with SM-2 beads (Bio-Rad) at 80 mg beads per 1 ml of liposome suspension (2 h at 23°C twice, overnight at 23°C once, and 2 h at 23°C once). Before use, SM-2 beads were washed in methanol, rinsed thoroughly with distilled water, and equilibrated in the liposome internal buffer. After detergent removal, proteoliposomes were concentrated to 50 mg/ml by ultracentrifugation at 86,000 *g* for 40 min at 4°C, freeze–thawed three times, and extruded through 400-nm membranes 10 times.

Uptakes were initiated by diluting reconstituted proteoliposomes 1:100 in the appropriate reaction buffer preincubated at 30°C. At the indicated time points, 200-μl reaction aliquots were removed and diluted in 2 ml of ice-cold quenching buffer (20 mM HEPES/Tris, pH 7, and 200 mM LiCl). The quenched reaction was immediately filtered through a 0.22-µm filter membrane (Millipore Sigma) and washed three times with 2 ml quenching buffer. Washed membranes were inserted into scintillation vials, and the membranes were soaked overnight in 5 ml Econo-Safe Counting Cocktail. Radioactivity in liposomes was measured using an LS6500 scintillation counter (Beckman Coulter).

### Isothermal titration calorimetry (ITC)

Substrate-free P-Glt_Ph_ and Glt_Ph_ proteins were affinity purified, thrombin cleaved, and purified by SEC in 20 mM HEPES/KOH, pH 7.4, 99 mM potassium gluconate, 1 mM sodium gluconate, and 1 mM DDM. Proteins were immediately concentrated to >5 mg/ml and diluted 2.5-fold to a final concentration of 30–50 µM. When diluted, the sample was supplemented with a final concentration of 1 mM DDM, 58 mM HEPES/KOH, pH 7.4, and an appropriate amount of sodium salt. 350 μl of protein samples was degassed and equilibrated to the temperature of the experiment, and ∼300 μl was loaded into the reaction cell of a small-volume NanoITC (TA Instruments), or in the case of TFB-TBOA experiments, an Affinity Auto ITC (TA Instruments). Titrant was prepared in a buffer matching the reaction cell, except that it contained the appropriate amount of L-Asp (A52100; RPI) and no protein or DDM. Dilution of DDM in the reaction cell over the course of the experiment had negligible effects on the injection heats, as previously noted (Boudker and Oh, 2015). 2 μl of titrant was injected every 5–6 min, at a constant temperature and a stirring rate of 250 rpm (125 rpm for TFB-TBOA experiments). Injection heats measured after protein was saturated with the ligand were used to determine the dilution heats subtracted from the ligand binding heats. Data were analyzed using NanoAnalyze software (TA Instruments) applying either the “independent” or “multiple sites” (referred to as “single-state” and “two-state,” respectively, throughout the text) binding models. For two-state binding, where each state is independent and nonidentical, the binding polynomial can be expressed as

$$\Sigma =1+K_{1}\left[S\right]+K_{2}\left[S\right]+K_{1}K_{2}{\left[S\right]}^{2},$$

where *K*_*i*_-s are the binding constants and [*S*] is the concentration of free L-Asp. The fraction of total protein bound is given by the following:

$$\frac{\left[PS\right]}{\left[P\right]}=\frac{n_{1}K_{1}\left[S\right]}{1+K_{1}\left[S\right]}+\frac{n_{2}K_{2}\left[S\right]}{1+K_{2}\left[S\right]},$$

where *n*_*i*_ is the apparent number of sites per protein molecule.

For the TFB-TBOA competition experiments, protein was first titrated with TBOA to saturation. Then, the concentration of TFB-TBOA was increased to a final concentration of 150 μM of TFB-TBOA, so that the overflow protein was also saturated. The appropriate amount of L-Asp was subsequently titrated into the saturated protein to yield a binding curve.

### Cryo-EM imaging data collection

In both data sets, 3.5 μl protein at ∼4.5 mg/ml was applied to a glow-discharged QuantiFoil R 1.2/1.3, 300-mesh, gold grid (Electron Microscopy Sciences). Grids were blotted at room temperature and 100% humidity for 3 s at 0 blot force and plunge frozen in liquid ethane using a VitroBot Mark IV (FEI). Data S1 was collected on P-Glt_Ph_, which was affinity-purified, concentrated, and buffer-exchanged into 20 mM HEPES/Tris, pH 7.4, 99 mM K-gluconate, 1 mM Na-gluconate, and 0.8 mM DDM to remove the substrate. The protein was then SEC purified in 20 mM HEPES/Tris pH 7.4, 250 mM NaNO_3_, and 0.8 mM DDM. 2.5 μl of substrate-free protein was applied to the grid, then 1 μl of L-Asp was added and mixed just prior to freezing so that the final substrate concentration was 1 mM, the final protein concentration was ∼4.5 mg/ml, and protein was exposed to the substrate for ∼5 s prior to freezing (including blot time). Data S2 was collected on P-Glt_Ph_ SEC purified in 20 mM HEPES/Tris, pH 7.4, 250 mM NaNO_3_, 1 mM L-Asp, and 0.8 mM DDM. The final buffer conditions of Data S2 were identical to Data S1.

All imaging data were collected on Titan Krios microscopes (FEI) operated at 300 kV. Data S1 was collected on a K2 Summit direct electron detector (Gatan). Automated data collection was performed in counting mode using Leginon software (Suloway et al., 2005), with a magnification of 22,500×, electron exposure of 70.23 e^−^/Å^2^, 50 frames/s, a defocus range of −1.0 to −2.0 μm, and pixel size of 1.073 Å. Data S2 was collected with a K3 Summit direct electron detector (Gatan). Automated data collection was performed in superresolution counting mode using SerialEM software (Mastronarde, 2005) with a magnification of 81,000×, electron exposure of 47.91 e^−^/Å^2^, 30 frames/s, defocus range of −0.5 to −2.5 μm, and pixel size of 0.53 Å.

### Image processing

The frame stacks were motion-corrected using MotionCor2 (Zheng et al., 2017), with 2× binning in the case of Data S1, and contrast transfer function (CTF) estimation was performed using CTFFIND 4.1 (Rohou and Grigorieff, 2015). All datasets were processed using cryoSPARC 3.0 and Relion 3.0.8 simultaneously with default parameters unless otherwise stated (Su et al., 2020; Punjani et al., 2017; Zivanov et al., 2018). Specific information on the processing of each dataset is in Figs. S5 and S7. In brief, particles were nonspecifically picked from micrographs using the Laplacian of Gaussian (LoG) picker, aiming for ∼2,000 picks per micrograph. These particles were extracted at a box size of 240 pixels with 4× binning and then imported to cryoSPARC. The particles underwent one round of 2-D classification to remove artifacts, and then multiple rounds of heterogeneous refinement (C1) using eight total classes, seven of which were noisy volumes (created by one iteration of ab initio) and one was an unmasked 3-D model obtained from a previous processing pipeline. Once >95% of particles converged on a single class, the particles were converted back to Relion format via PyEM (Asarnow et al, 2019) and re-extracted at full box size. These particles were reimported to cryoSPARC, underwent three more rounds of heterogeneous refinement, then nonuniform (NU) refinement using C3 symmetry (dynamic mask threshold, dynamic mask near, and dynamic mask far were always set to 0.8, 12, and 28, respectively; Punjani et al., 2020). These particles were converted back to Relion format and underwent Bayesian polishing, using parameters obtained using 5,000 random particles within the set. After three more rounds of heterogeneous refinement in cryoSPARC and one round of NU-refinement, we performed local CTF refinement (minimum fit res 7 Å). After three more rounds of heterogeneous refinement and one round of NU-refinement, we performed global CTF refinement (three iterations, minimum fit res 7 Å; fitting trefoil, spherical aberration, and tetrafoil), three more rounds of heterogeneous refinement, and one round of NU-refinement.

Data S2 was also further processed to obtain the high-resolution structure by three additional rounds of polishing, local CTF refinement, and global CTF refinement as described above. During the polishing rounds, the box size and pixel size were rescaled as indicated in the supplement. After the second round of polishing, we classified single protomers by employing the relion_particle_symmetry_expand function in Relion to artificially expand the particle set three times (C3) so that each protomer rotated to the same position (Scheres, 2016). The expanded particle set was subjected to 3-D classification without alignment with T = 400 and 10 classes, using the refined C3 structure as a reference map. The exceptionally high T value was chosen to separate out subtle structural differences, and lower T values were also tested during processing. The local mask was created using a 20 Å map of the transport domain of Chain A of PDB accession no. 2NWX, with an initial binarization threshold of 0.01, extended by 3 pixels, and a soft-edge of 10 pixels. Of these, particle stacks from subsets of interest were separately used in cryoSPARC’s local refinement (C1), using the mask and map obtained from the most recent NU refinement. Single protomers in Data S1 were classified similarly, except we performed symmetry expansion after the first round of polishing due to the limited resolution of the dataset. After processing, the resulting half-maps were used as inputs for density modification implemented in PHENIX 1.19.1–4122 (Terwilliger et al., 2020; Adams et al., 2010), using a mask created from the NU-refinement job (threshold 0.1, dilation radius 15, soft padding width 5). All density maps were displayed using ChimeraX (Pettersen et al., 2021).

### Model building and refinement

For atomic model building, the crystal structure of WT Glt_Ph_ in the OFS (PDB accession no. 2NWX) was docked into the densities using UCSF Chimera (Pettersen et al., 2004). The model was first real-space refined in PHENIX (Adams et al., 2010). Then, chain A was adjusted manually, and ions were added in COOT (Emsley and Cowtan, 2004). Waters were initially added using phenix.douse, and subsequently manually inspected and adjusted. The resulting model underwent additional rounds of real-space refinement and validated using MolProbity (Chen et al., 2010). All structural models were displayed using ChimeraX (Pettersen et al., 2021). Per-residue Cα RMSDs were generated with Chimera (Meng et al., 2006). To cross-validate models, refined models (FSC_sum_) were randomly displaced an average of 0.3 Å using phenix.pdbtools. The displaced model was real-space refined against half-map 1 obtained through density modification to obtain FSC_work_. The resulting model was validated against half-map 2 to obtain FSC_free_.

### Single-molecule dynamics assay

Thrombin-cleaved P-Glt_Ph_, containing C321A and N378C mutations, was labeled and reconstituted as described previously (Akyuz et al., 2013). Protein was SEC purified in 20 mM HEPES/Tris, 200 mM NaCl, 1 mM L-Asp, and 1 mM DDM. Purified protein was labeled using maleimide-activated LD555P-MAL and LD655-MAL dyes and biotin-PEG_11_ at a molar ratio of 4:5:10:2.5. Excess dyes were removed on a PDMiniTrap Sephadex G-25 desalting column (GE Healthcare).

All experiments were performed on a previously described home-built prism-based TIRF microscope constructed around a Nikon Eclipse Ti inverted microscope body (Juette et al., 2016). Microfluidic imaging chambers were passivated with biotin-PEG, as previously described (Akyuz et al., 2015). After passivation, the microfluidic channel was incubated with 0.8 μM streptavidin (Invitrogen) in T50 buffer (50 mM NaCl and 10 mM Tris, pH 7.5) for 7 min, then thoroughly rinsed with T50 buffer. Detergent-solubilized protein was immobilized by slowly flowing over the channel, and excess protein was removed by washing with 1 ml of 25 mM HEPES/Tris, pH 7.4, 200 mM KCl, and 1 mM DDM.

Buffers were supplemented with an oxygen-scavenging system composed of 2 mM protocatechuic acid and 50 nM protocatechuate-3,4-dioxygenase, as described previously (Aitken et al., 2008). The smFRET movies were recorded with 100-ms integration time using 80–100 mW laser power. All conditions tested were in the presence of 25 mM HEPES/Tris, 500 mM sodium salt, and 1 mM DDM, in the presence or absence of 1 mM L-Asp. Slides were washed with 25 mM HEPES/Tris, 200 mM KCl, and 1 mM DDM between experiments. Traces were analyzed using Spartan software (Juette et al., 2016). Trajectories were corrected for spectral cross talk and preprocessed automatically to exclude trajectories that lasted ≤15 frames and had a signal-to-noise ratio of ≤8. Traces with multiple photobleaching events (indicative of multiple sensors in a protein) or inconsistent total fluorescence intensity were also discarded.

### Single-molecule transport assay and analysis

Thrombin-cleaved Glt_Ph_ (C321A/N378C) was SEC purified in 20 mM HEPES/Tris, pH 7.4, 200 mM NaCl, and 0.1 mM L-Asp. Protein was labeled with maleimide-activated biotin-PEG_11_ (EZ-Link; Thermo Fisher Scientific) in the presence of *N-*ethylmaleimide at a molar ratio of 1:2:4 as previously described (Ciftci et al., 2020). Liposomes were prepared from a 3:1 (wt/wt) mixture of *E. coli* polar lipid extract (Avanti Polar Lipids) and egg phosphatidylcholine in SM-KCl buffer (50 mM HEPES/Tris, pH 7.4, and 200 mM KCl). Liposomes were extruded through 400-nm filters (Whatman Nucleopore) using a syringe extruder (Avanti), and destabilized by the addition of Triton X-100 at 1:2 (wt/wt) detergent-to-lipid ratio. Labeled Glt_Ph_ was added to the liposome suspension at 1:1,000 (wt/wt) protein-to-lipid ratio at room temperature for 30 min. Detergent was removed by six rounds incubation of Bio-Beads (two rounds at 23°C for 2 h each, one round at 4°C overnight, and three rounds at 4°C for 2 h each). The excess substrate was removed by three rounds of centrifugation for 1 h at 49,192 *g* at 4°C, removal of the supernatant, addition of 1 ml fresh SM buffer, and three freeze/thaw cycles. Liposomes were concentrated to 50 mg/ml, and ccPEB1a-Y198F labeled with activated LD555P-MAL and LD655-MAL dyes as described (Ciftci et al., 2020) was added at a final concentration of 0.6 μM and encapsulated by two freeze–thaw cycles. To remove unencapsulated ccPEB1a-Y198F, 1 ml of SM-KCl buffer was added, and liposomes were centrifuged for 1 h at 49.192 *g* at 4°C. Supernatants were discarded, and liposomes were suspended at 50 mg/ml and extruded 12 times through 100-nm filters.

Single-transporter smFRET assays were performed on the same microscope described above, and the microfluidic imaging chambers were prepared in the same way. After coating with streptavidin, the channel was rinsed thoroughly with SM-K(X) buffer (50 mM HEPES/Tris, pH 7.4, containing 200 mM potassium salt buffer, where the anion (X) was changed based on the condition tested). Extruded liposomes were immobilized by slowly flowing over the channel, and excess liposomes were removed by washing with 1 ml SM-K(X) buffer.

Buffers were supplemented with an oxygen-scavenging system as above, and the smFRET movies were recorded with 400-ms integration time using 20–40-mW laser power. To confirm that liposomes were not leaky, a video was taken in SM-K(X) buffer containing 1 μM L-Asp and 1 μM valinomycin. No L-Asp uptake was observed under these conditions lacking Na^+^ gradient. After completion of this video, another video was initiated to record transport events. At ∼3 s into this video, SM-Na(X) buffer containing 1 μM L-Asp and 1 μM valinomycin was perfused into the channel.

Traces were analyzed using Spartan software (Juette et al., 2016). Trajectories were corrected for spectral cross talk and preprocessed automatically to exclude trajectories that lasted ≤15 frames, had a signal-to-noise ratio of ≤8, and had an initial FRET efficiency <0.4 or >0.7. Traces with multiple photobleaching events (indicative of multiple sensors in a liposome) or inconsistent total fluorescence intensity were also discarded. Remaining traces were sorted by either the presence or absence of observable transport events (responding and nonresponding traces, respectively), determined by an increase in FRET efficiency from ∼0.55–0.6 to ∼0.75–0.8.

Responding traces from each video were plotted as time-dependent mean FRET efficiency. Buffer replacement time was determined as described (Ciftci et al., 2020). Within each dataset, the data were normalized so that 0% was the first time point and 100% was the first time point +0.2 (the change in FRET efficiency upon saturation of ccPEB1a-Y198F). The resulting normalized data were multiplied by the fraction of the responding traces relative to the total traces. Data from three independent reconstitutions were merged and fitted to a triexponential function in GraphPad Prism 8.4.2, where Y0 = 0, plateau = 1, percentages were set between 0 and 100, and all rate constants were set to be shared between all data sets and >0.

### Online supplemental material

**Figure S1.**
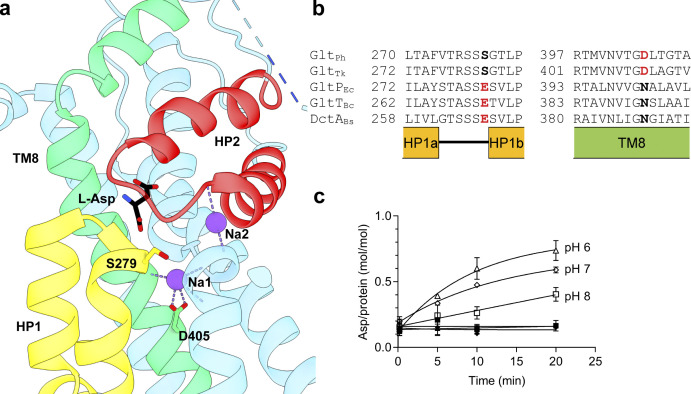
**P-Glt**_**Ph**_ **(S279E/D405N) has partial proton dependence. (a)** Structural model of substrate-bound Glt_Ph_ (PDB accession no. 2NWX). **(b)** Sequence alignment of Na^+^-coupled (Glt_Ph_ and Glt_Tk_) and H^+^-coupled (GltP_Ec_, GltT_Bc_, and DctA_Bs_) transporters; structural elements are indicated below the alignment. Residues mutated in P-Glt_Ph_ are in bold. **(c)** pH-dependent aspartate uptake of P-Glt_Ph_. Proteoliposomes were loaded with 50 mM potassium phosphate buffer, pH 7, and 100 mM potassium acetate and diluted into the following 50 mM buffers containing 1 μM [^3^H]L-Asp: MES/NMDG, pH 6 (triangles); HEPES/Tris, pH 7 (diamonds); or HEPES/Tris, pH 8 (squares). Buffers contained either 100 mM KCl (filled symbols) or 100 mM NaCl (empty symbols). Solid lines are shown to guide the eye, and error bars (SD) not displayed represent errors smaller than the size of the symbol.

**Figure S2.**
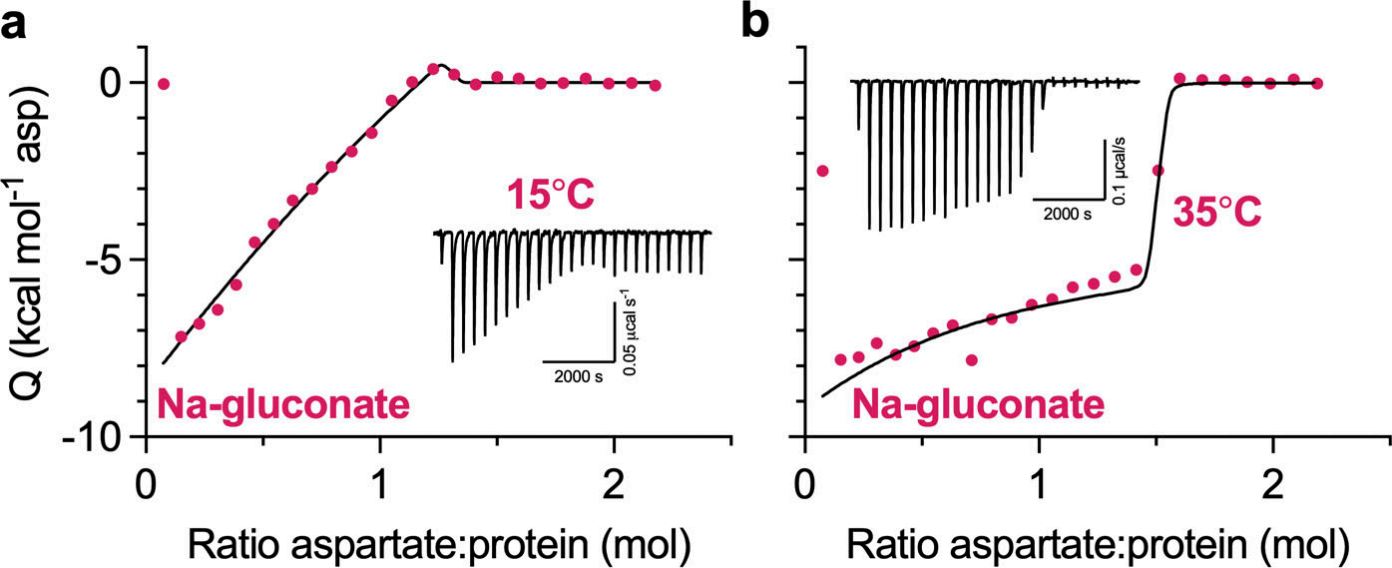
**Simulated fits of WT binding isotherms. (a)** WT Glt_Ph_, 500 mM Na-gluconate, 15°C; k_D_1 and k_D_2 of Fit 1 (solid) and Fit 2 (dashed) are constrained to 0.3 and 0.4 nM, respectively. Fit 1 and Fit 2 have parameters of *n*_*1*_, 0.82 and 0.37; *ΔH*_*1*_, −22.7 and −50.0 kcal mol^−1^; *n*_*2*_, 0.36 and 0.89; *ΔH*_*2*_, 39.4 and 16.2 kcal mol^−1^. **(b)** WT Glt_Ph_, 500 mM Na-gluconate, 35°C; k_D_1 and k_D_2 are constrained to 1.6 and 3.6 nM, respectively. Fit has parameters of *n*_*1*_, 0.16; *ΔH*_*1*_, −24.6 kcal mol^−1^; *n*_*2*_, 1.30; *ΔH*_*2*_, −4.7 kcal mol^−1^.

**Figure S3.**
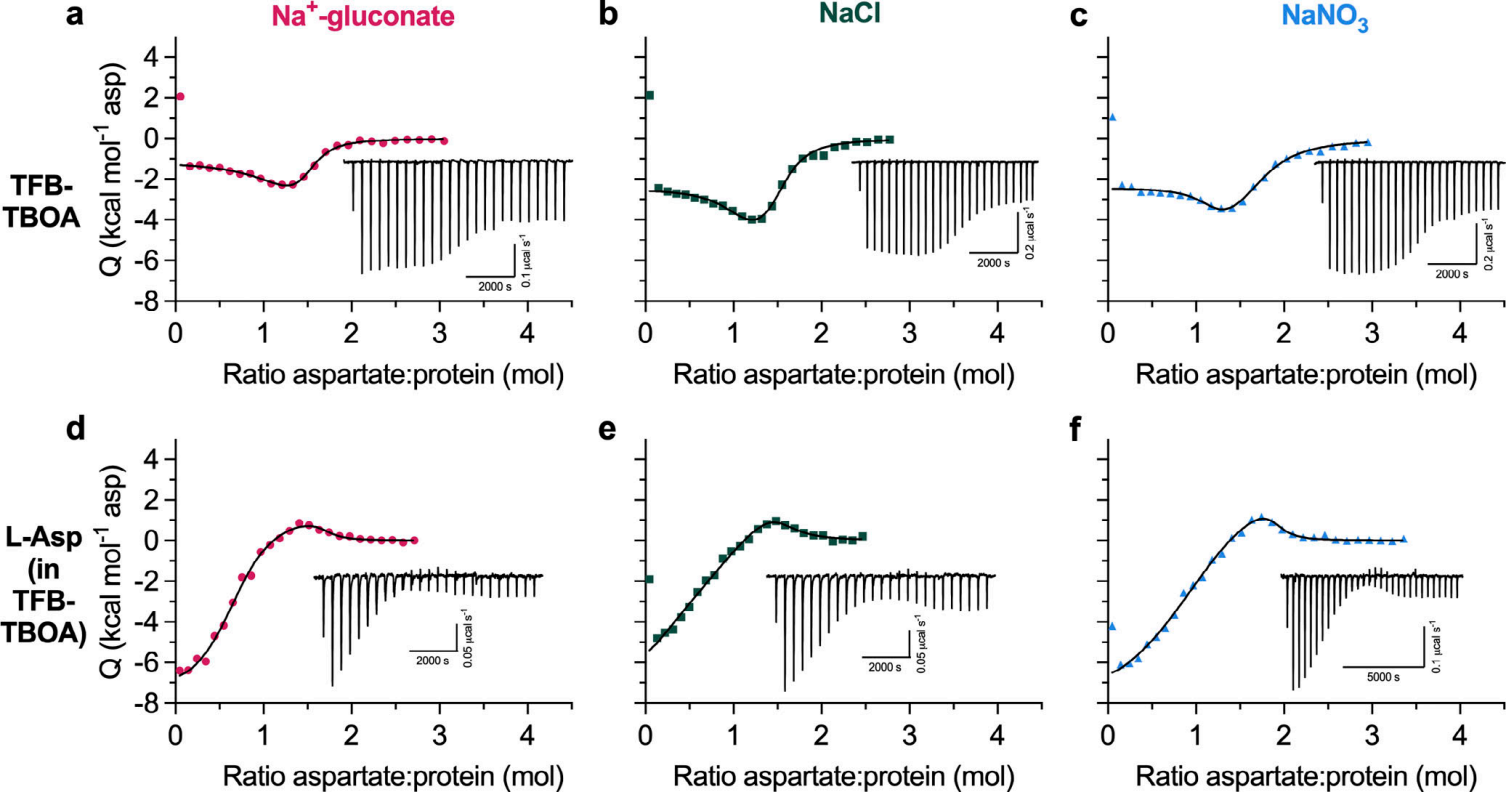
**TFB-TBOA binds to two states in WT Glt**_**Ph**_**.** All ITC experiments were performed at 15°C in buffers containing 500 mM Na-gluconate (red circles), NaCl (green squares), or NaNO_3_ (blue triangles). Insets show the thermal power with the corresponding scales. All data were fitted to the two-state model; however, exact binding parameters cannot be reliably determined. **(a–c)** TFB-TBOA binding isotherms. **(d–f)** Aspartate competition isotherms in the presence of saturating TFB-TBOA concentrations (see Materials and methods).

**Figure S4.**
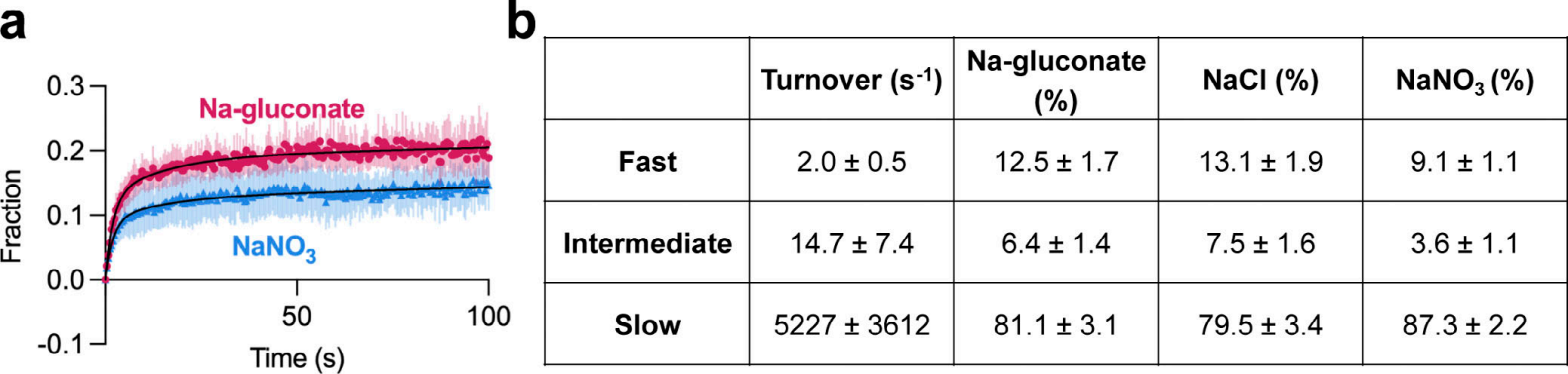
**Fitted parameters of L-Asp uptake by WT Glt**_**Ph**_ **in the presence of various anions. (a)** Aspartate transport of Glt_Ph_ (C321A/N378C) was measured using the single-transporter FRET-based assay in NaNO_3_ (blue) or Na-gluconate (red). Data are means and SE from three independent experiments performed as in Fig. 2 f. **(b)** Data in a and Fig. 2 f were fitted to triexponential functions (a three-phase association model; GraphPad Prism). Initial and final fractions of total possible uptake were set to 0 and 1, respectively. The three turnover rates (fast, intermediate, and slow), corresponding to the heterogeneous transporter populations, were constrained to be the same for all datasets (see Materials and methods for a detailed description of data processing). Three independent experiments per condition were analyzed, and values shown are means and SEM of the fitted parameters.

**Figure S5.**
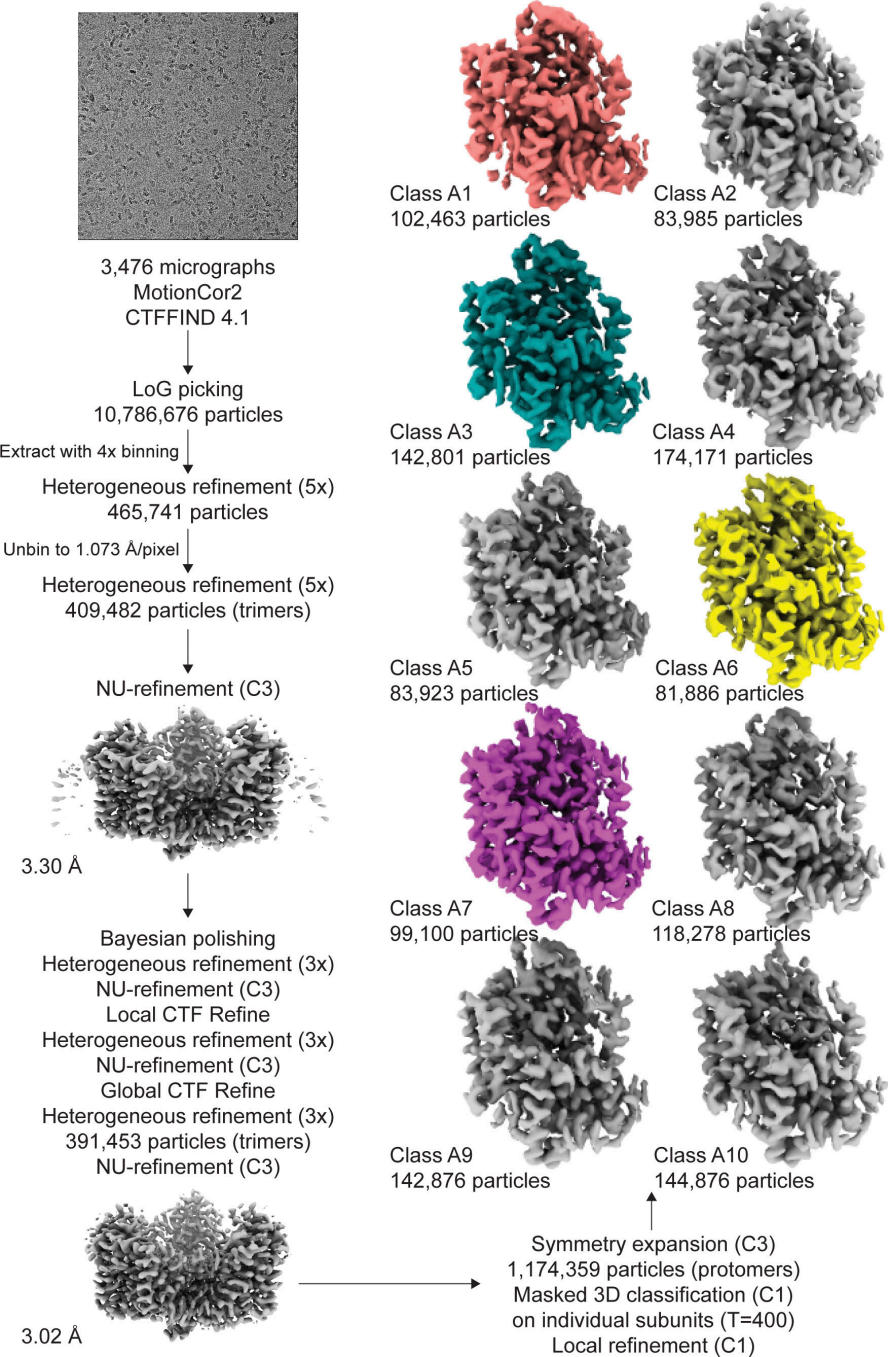
**Processing flowchart for Data S1.** Protomer maps from masked classification are unsharpened, with the two other protomers removed for clarity. All maps are contoured at 8 σ.

**Figure S6.**
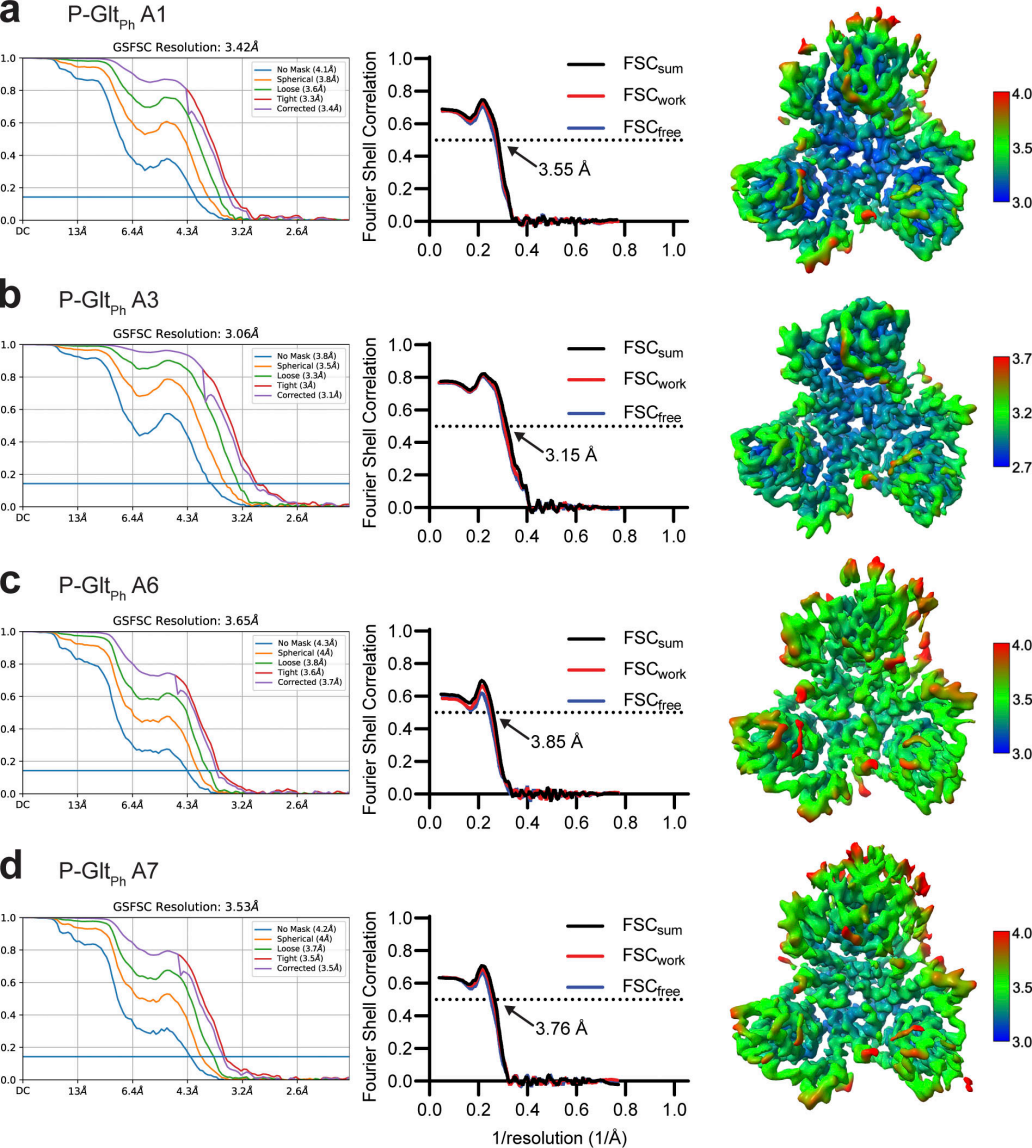
**Validation statistics for models from Data S1.** From left to right, map FSC from NU-refinement in cryoSPARC, model-to-data validation in Phenix of the single protomer, and local resolution estimation of the unsharpened map. All maps are contoured at 8 σ. The top protomer is the subject of focused classification and model refinement.

**Figure S7.**
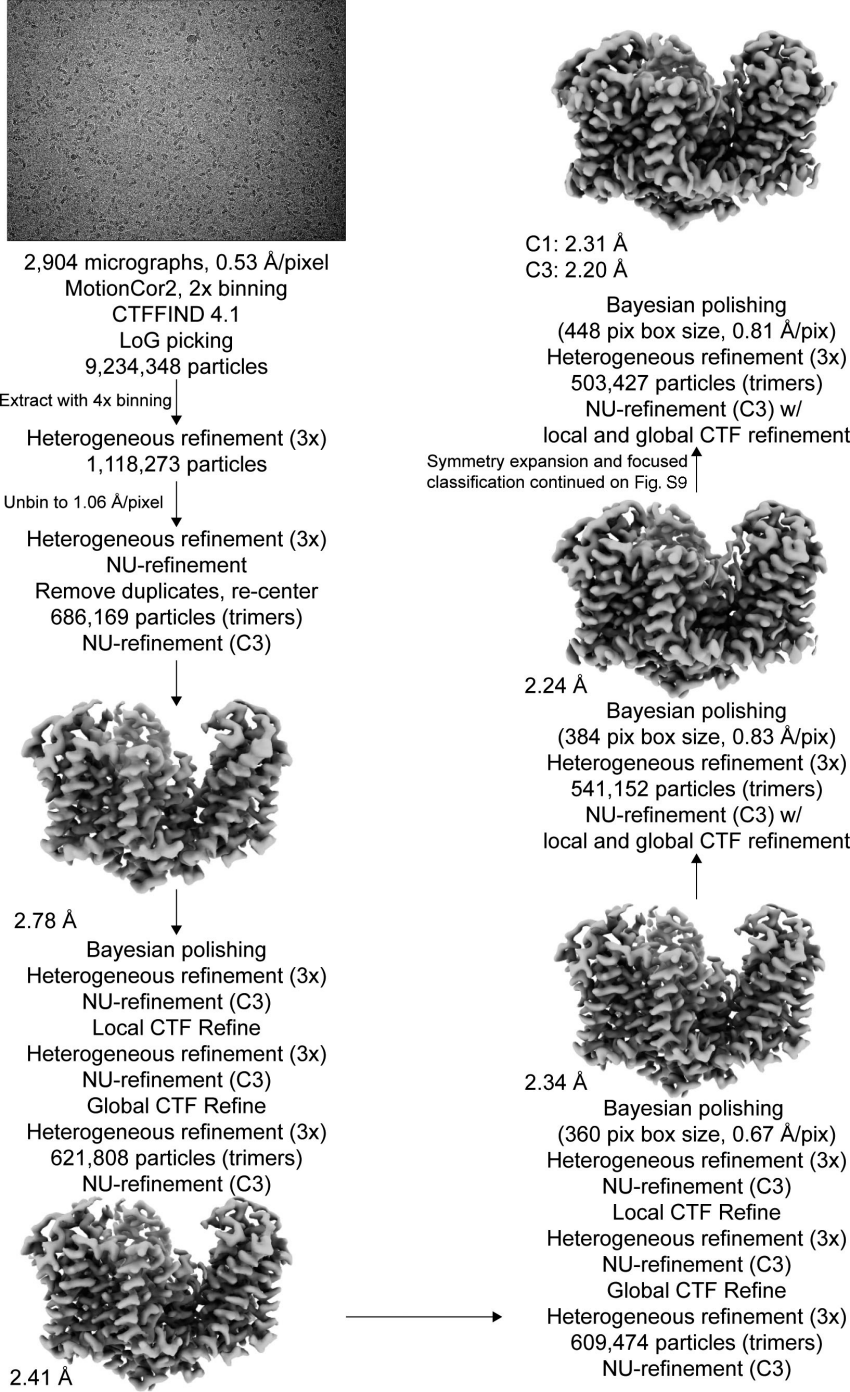
**Processing flowchart for Data S2.** Unsharpened maps are contoured at σ of 8.

**Figure S8.**
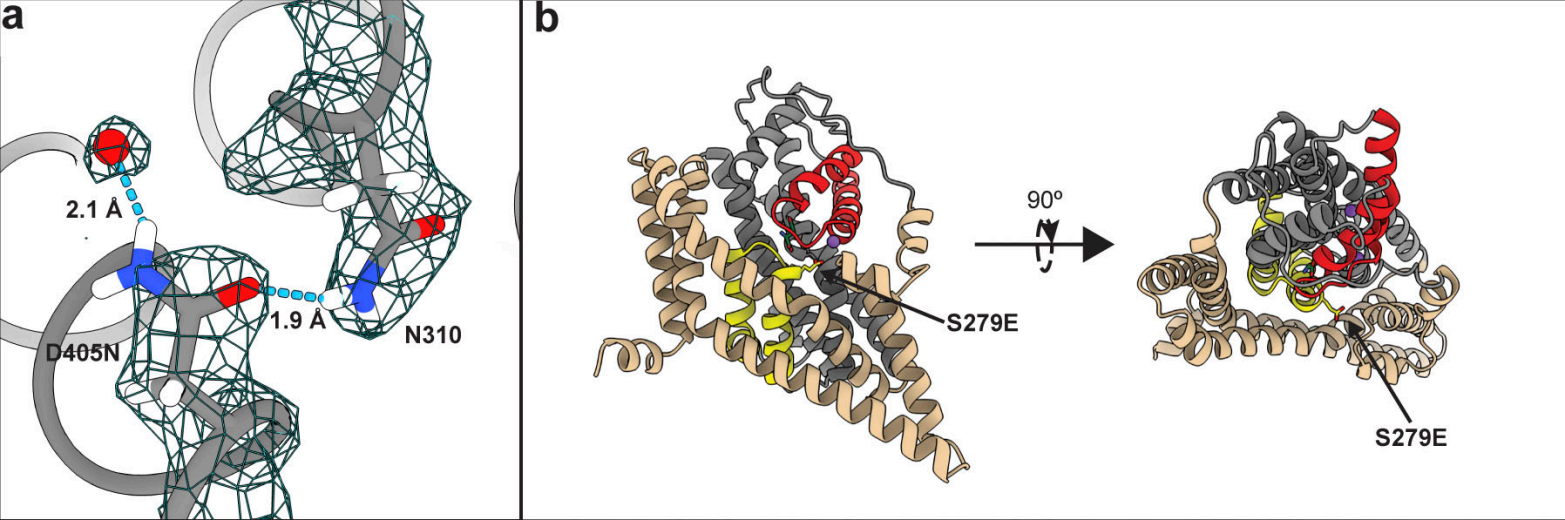
**Effects of mutations on P-Glt**_**Ph**_**. (a)** Close-up view of S279 mutation. The model and density map are from Data S2 refined in C3 (PDB accession no. 7RCP). A water molecule replacing Na1 and coordinated by D405N is emphasized as a red ball. Hydrogen bonds are displayed using hbond in ChimeraX. **(b)** Cartoon representation of a protomer viewed in the membrane plane (left) and from the extracellular space (right), showing S279E points away from the transport domain.

**Figure S9.**
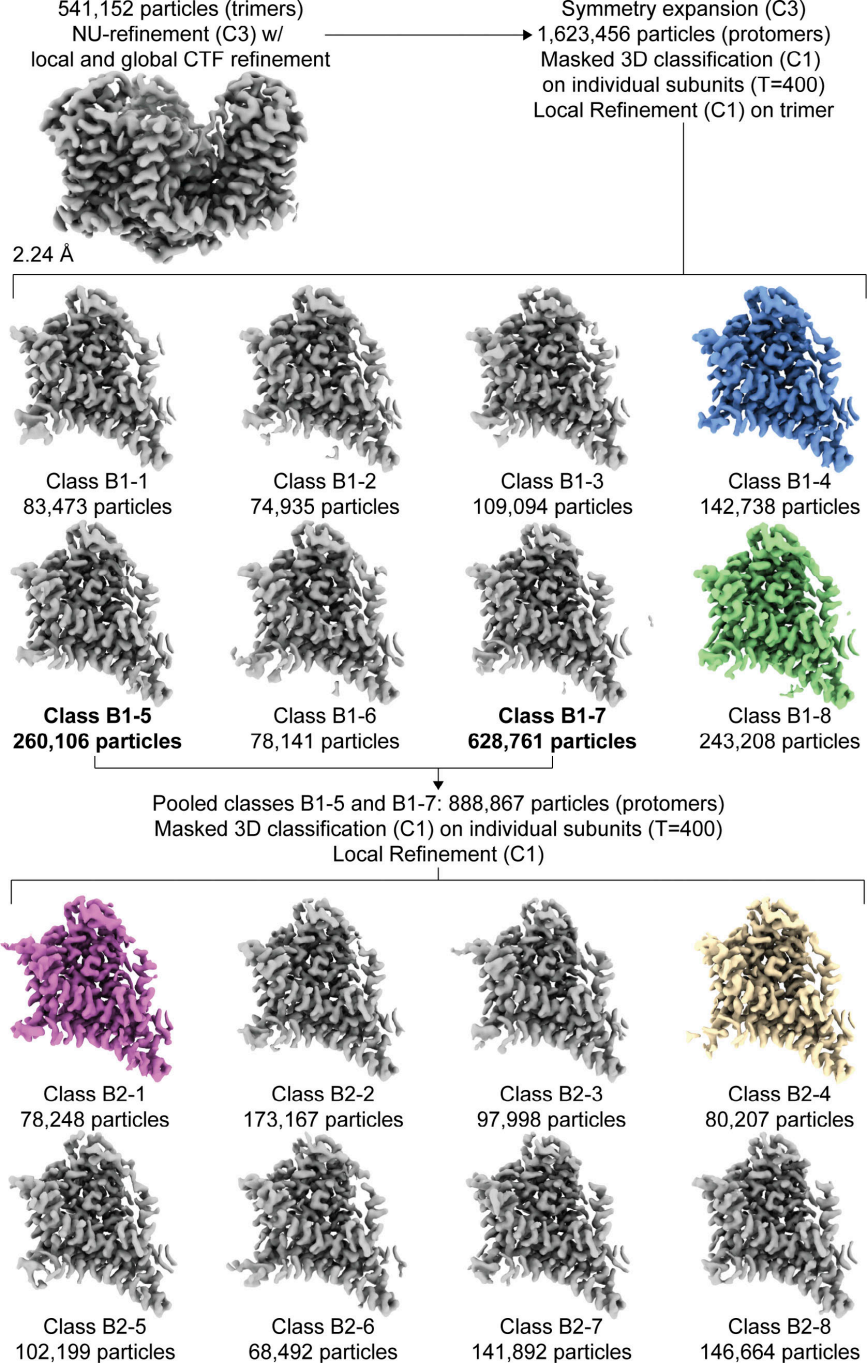
**Focused classification of Data S2.** Protomer maps from masked classification are unsharpened, with the two other protomers removed for clarity. Colored classes were used for further model refinement (blue, OFS_in_; green, OFS_mid_; purple, OFS_out_, D390 down; wheat, OFS_out_, D390 up). All maps are contoured at σ of 8.

**Figure S10.**
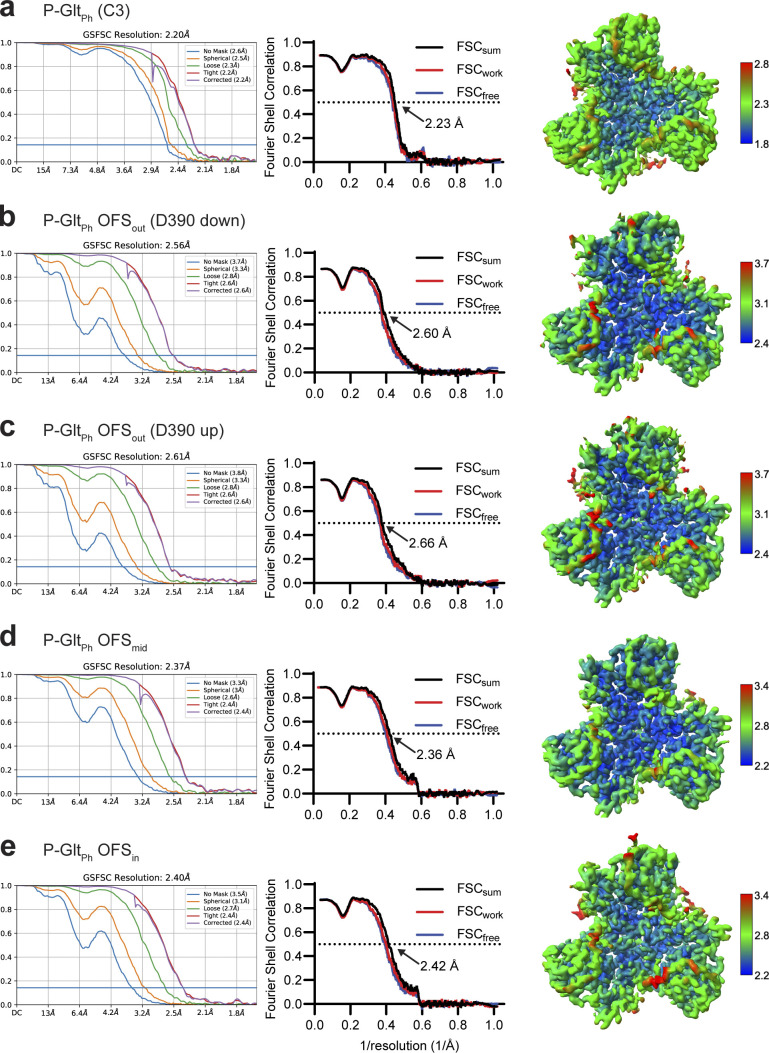
**Validation statistics for models from Data S2. (a–e)** OFS-C3 (a); OFS_out_, D390 down (b); OFS_out_, D390 up (c); OFS_mid_ (d); OFS_in_ (e). From left to right, map FSC from NU-refinement in cryoSPARC, model-to-data validation in Phenix of the trimer, and local resolution estimation. All maps are contoured at 8 σ except a, which is contoured at 10 σ. The top protomer in b–e is the subject of focused classification and model refinement.

**Figure S11.**
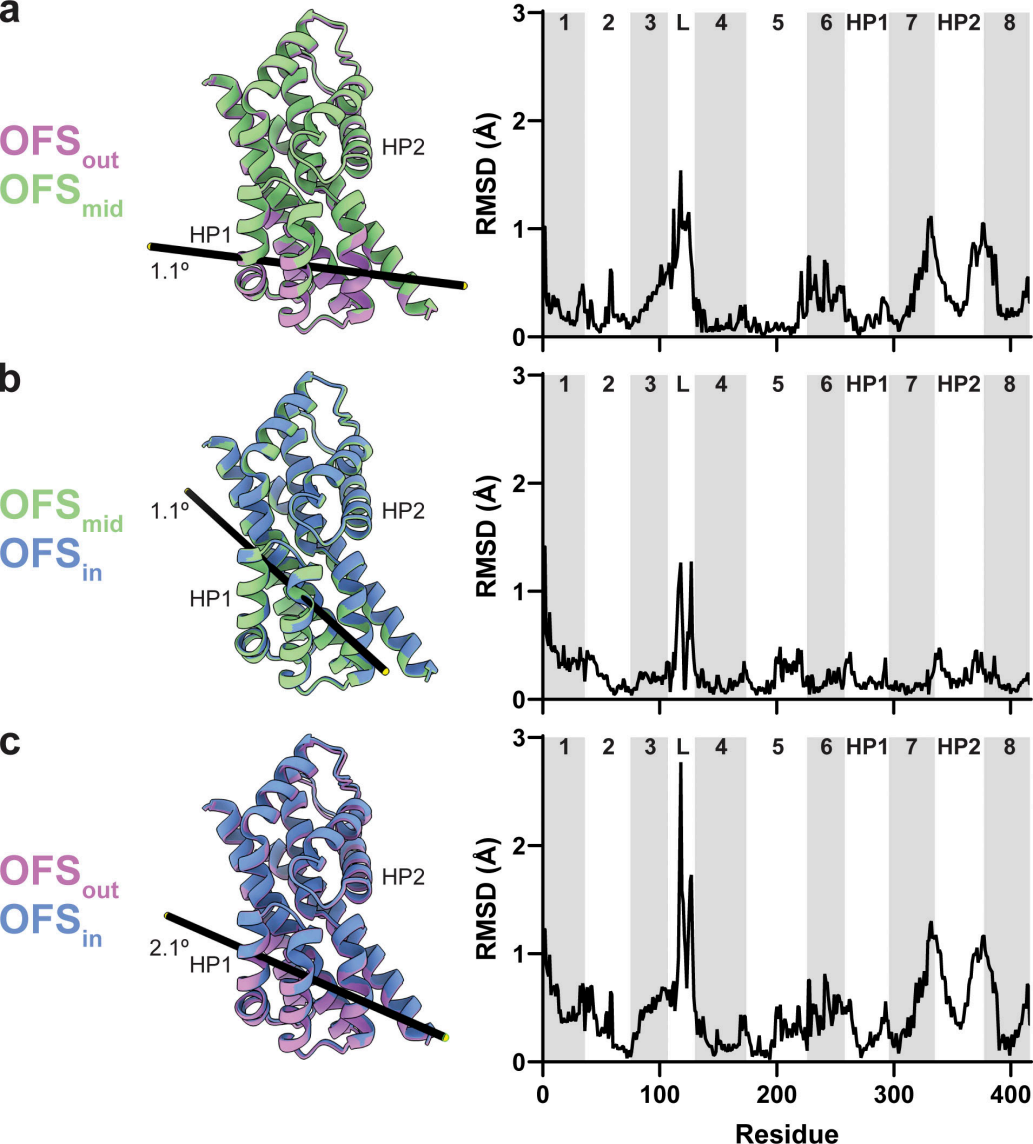
**Protomer tilts in the OFS.** Trimers were superimposed on the trimerization regions (residues 150–195) of all three protomers. **(a–c)** Comparison of the tilts for OFS_out_/OFS_mid_ (a); OFS_mid_/OFS_in_ (b); and OFS_out_/OFS_in_ (c). OFS_out_, OFS_mid_, and OFS_in_ are purple, green, and blue, respectively. Although parts of the scaffold domain also move (see Videos 2, 3, and 4), only transport domains of the classified protomers are shown for clarity. Black bars represent tilt axes and angles calculated using the align command in ChimeraX. The corresponding per-residue Cα RMSDs are on the right. Transmembrane domains are labeled and alternatively shaded. L, the flexible loop between TM3 and TM4.

**Figure S12.**
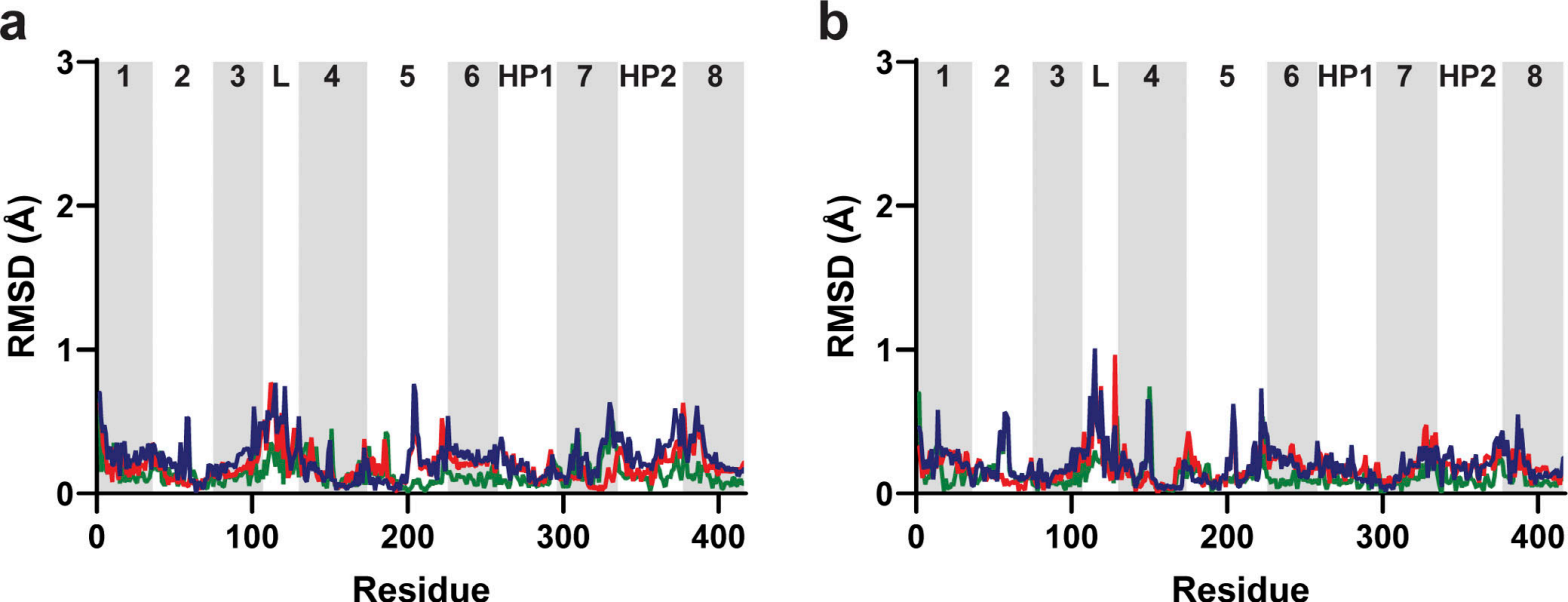
**Protomers adjacent to chain A do not display concerted movements.** Trimers were aligned along the trimerization regions of all three protomers (residues 150–195). **(a and b)** Per-residue Cα RMSDs of chain B (a) and chain C (b). Lines are OFS_out_/OFS_mid_ (green); OFS_mid_/OFS_in_ (red); and OFS_out_/OFS_in_ (blue). Transmembrane domains are labeled and alternatively shaded. L, the disordered loop between domains 3 and 4.

**Figure S13.**
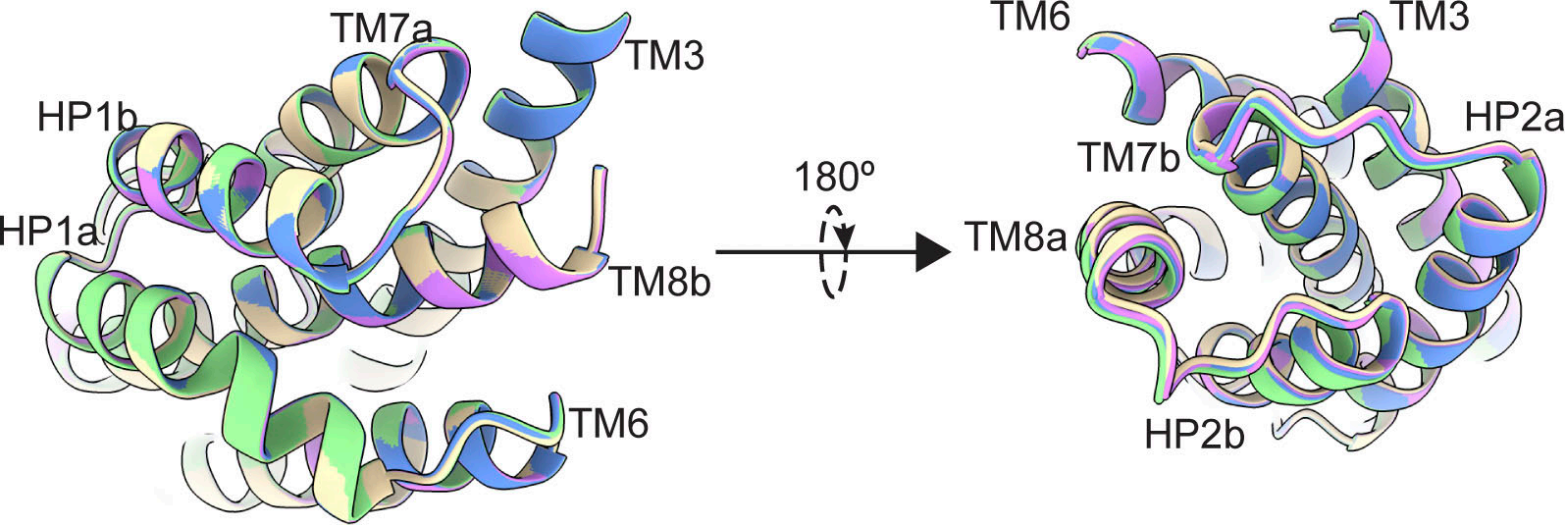
**P-Glt**_**Ph**_ **at equilibrium does not have mobility in extracellular helices.** Transport domains were superimposed on HP1 and TM7a (residues 258–309). Superimposition of transport domains from Data S2. OFS_out_ (D390 down) is purple, OFS_out_ (D390 up) wheat, OFS_mid_ green, and OFS_in_ blue. The views are from the intracellular (left) and extracellular (right) sides of the transport domain.

Fig. S1 shows the location of P-Glt_Ph_ mutations and their effect on function. Fig. S2 illustrates how complex ITC isotherms change depending on parameters. Fig. S3 shows binding of TFB-TBOA to WT Glt_Ph_. Fig. S4 shows the fitted parameters of WT Glt_Ph_ uptake in the presence of different anions. Figs. S5 and S6 are processing flowcharts and model validations of structures from Data S1. Figs. S7, S9, and S10 are processing flowcharts and map validations of structures from Data S2. Fig. S8 shows the effect of P-Glt_Ph_ mutations on structure. Fig. S11 shows the angles of protomer tilts between OFS_out_, OFS_mid_, and OFS_in_. Fig. S12 shows the changes in adjacent protomers in OFS_out_, OFS_mid_, and OFS_in_. Fig. S13 shows the conformation of extracellular helices in OFS_out_, OFS_mid_, and OFS_in_. Video 1 shows different viewing angles of Fig. 3 a. Videos 2 and 3 show 3-D variability (3DVA) components of OFS_out_/OFS_mid_ and OFS_mid_/OFS_in_, respectively. Video 4 shows structural transitions between OFS_out_, OFS_mid_, and OFS_in_. Tables S1 and S2 show fitted parameters of L-Asp binding to P-Glt_Ph_ at 10°C and 15°C, respectively. Tables S3 and S4 show model validations of Data S1 and S2, respectively. Table S5 shows structural differences between structures with packing heterogeneity and OFS_out_, OFS_mid_, and OFS_in_. Table S6 show the tilt states arising from 3-D classification of Data S2. Data S1 provides maps and models from P-Glt_Ph_ where substrate was added just before freezing. Data S2 provides maps and models from P-Glt_Ph_ where substrate was present throughout the purification.

## Results

### P-Glt_Ph_ (S279E/D405N) reveals two outward-facing substrate-binding conformations modulated by temperature and salts

**Figure 1.**
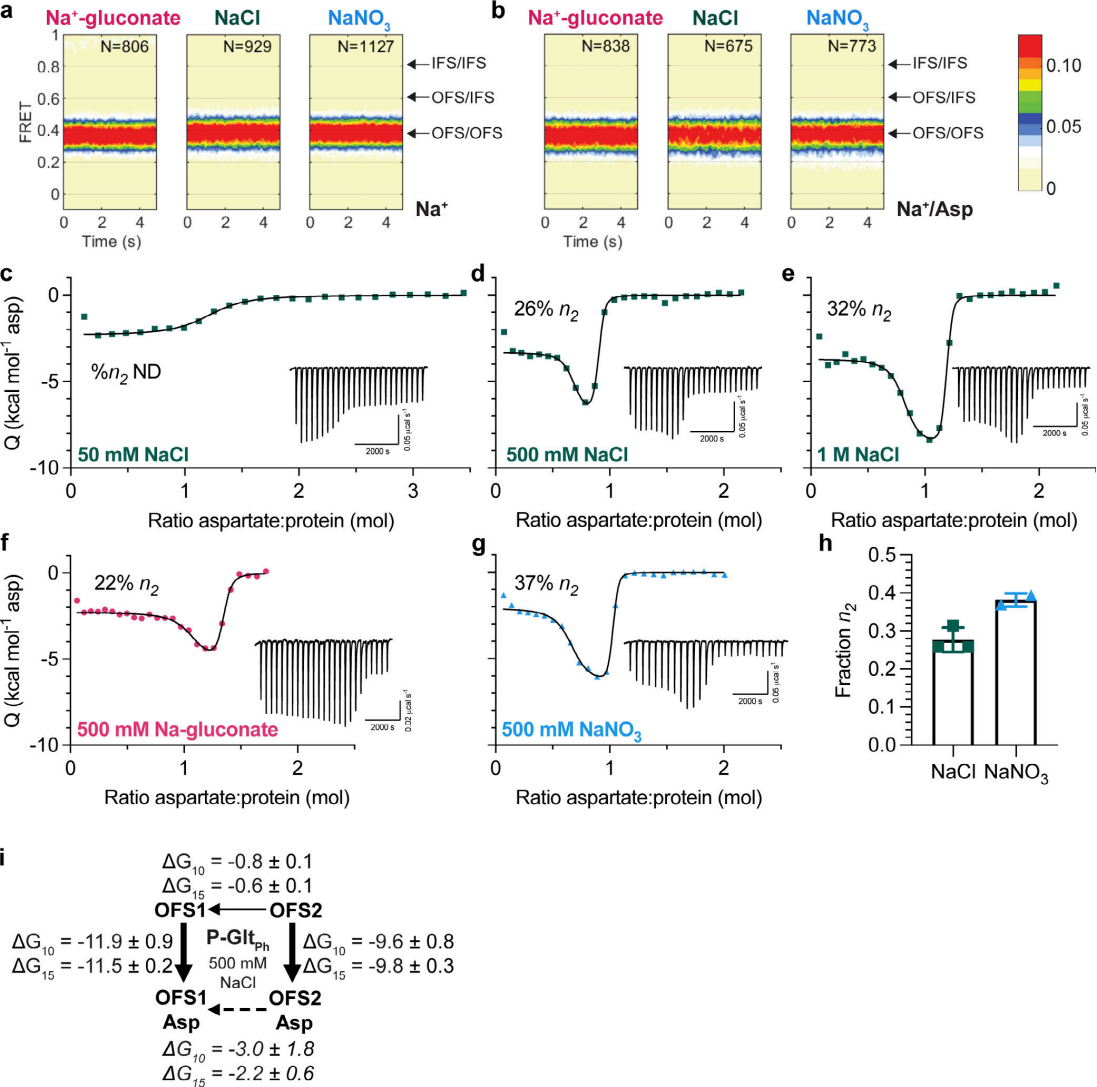
**Two outward-facing substrate-binding states in P-Glt**_**Ph**_ **(S279E/D405N). (a and b)** FRET efficiency population histograms of P-Glt_Ph_ in the presence of 500 mM sodium salts, in the absence (a) or presence (b) of 1 mM L-Asp. *N* is the number of molecules analyzed. Data shown are an aggregate of two independent experiments. Population contour plots are color-coded from tan (lowest population) to red (highest). Expected conformations according to *E*_*FRET*_ values are indicated by arrows. **(c–g)** ITC experiments on P-Glt_Ph_ at 15°C were performed at least twice on independently prepared protein samples with similar results. Insets show the thermal power with the corresponding scales. **(c–e)** Aspartate binding isotherms derived from the ITC experiments in the presence of different amounts of NaCl (green squares): 50 mM (c); 500 mM (d); and 1 M (e). The 50-mM data were fitted to the single-state model, with fitted *K*_*d*_ = 917 nM, Δ*H =* −2.3 kcal mol^−1^, and an apparent number of binding sites, *n* = 1.18. 500 mM NaCl and 1 M NaCl data were fitted to the two-state binding model. The 500-mM NaCl data gave the following fitted parameters for the two states: *K*_*d*_, 1.3 and 60.8 nM; Δ*H*, −3.3 and −7.1 kcal mol^−1^; *n*, 0.64 and 0.22. The 1-M NaCl data: *K*_*d*_, 0.5 and 27.4 nM; Δ*H*, −3.7 and −8.7 kcal mol^−1^; *n*, 0.77 and 0.38. **(f and g)** Aspartate binding isotherms were obtained in 500 mM Na-gluconate (f, red circles), or NaNO_3_ (g, blue triangles). All data were fitted to the two-state model. The 500-mM Na-gluconate data: *K*_*d*_, 4.4 and 138.3 nM; Δ*H*, −2.3 and −5.5 kcal mol^−1^; *n*, 1.00 and 0.28. The 500-mM NaNO_3_ data: *K*_*d*_, 0.9 and 34.3 nM; Δ*H*, −2.1 and −6.5 kcal mol^−1^; *n*, 0.62 and 0.37. **(h)** Comparison of the *n*_*2*_ fraction in 500 mM NaCl or NaNO_3_. Each point is an independent experiment. **(i)** Schematic representations of the conformational and binding equilibria obtained experimentally at 10°C and 15°C in 500 mM NaCl (solid lines) or inferred (dashed lines). The thermodynamic parameters were estimated under the assumptions that there are two nonexchanging binding states. Directions of the arrows indicate directions of the free energy changes, ∆*G*-s, shown. All values are in kilocalories per mole. Binding *∆G*-s are from Tables S1 and S2. The free energy differences between sodium-bound OFSs were calculated from equilibrium constants *K*_*eq*_ = *n*_1_/*n*_2_. Thin lines represent steps that are slow on the time scale of ITC experiments.

We generated a mutant, P-Glt_Ph_, that eliminates Na^+^ binding to Na1 (D405N) and introduces a protonatable residue at the tip of HP1 (S279E), mimicking amino acid sequence features of proton-coupled orthologues (Fig. S1, a and b). Our original intention was to test the pH dependence of this mutant. While proton gradients stimulated P-Glt_Ph_ activity in the presence of Na^+^ (Fig. S1 c), this is not the focus of this study. We observed that P-Glt_Ph_ had greatly diminished transport compared to the WT transporter (Ryan et al., 2009), prompting us to test its ability to translocate substrate using smFRET. We introduced a single cysteine mutation into a cysteine-free background (P-Glt_Ph_ C321A/N378C), purified the protein in DDM, labeled with donor and acceptor fluorophores, and analyzed by smFRET as in earlier studies (Akyuz et al., 2013; Akyuz et al., 2015; Huysmans et al., 2021). Conformations of protomer pairs within individual Glt_Ph_ trimers can be distinguished by FRET efficiency (*E*_*FRET*_) as either both in OFS (∼0.4), a mixture of OFS and IFS (∼0.6), or both in IFS (∼0.8). Most P-Glt_Ph_ molecules occupy a low *E*_*FRET*_ state in the presence of 500 mM sodium salts, regardless of anion or substrate presence (Fig. 1, a and b). Thus, this mutant is mainly in OFS or the intermediate iOFS (Huang et al., 2020; Verdon and Boudker, 2012), which our smFRET setup cannot distinguish.

Further characterization of substrate binding to the mutant yielded results incompatible with our current understanding of the glutamate transporter mechanism. We expected to observe simple 1:1 binding of aspartate to a single P-Glt_Ph_ binding site in ITC experiments. Instead, we observed bimodal binding isotherms in 500 mM NaCl at 15°C (Fig. 1 d). Because this mutant exclusively occupies OFS, this result suggests heterogeneity in substrate binding to this state. A two-state model assuming two independent, nonidentical binding states (OFS1 and OFS2; Freire et al., 2009) is the simplest model that fits this data reliably. Several other binding models for complex equilibria, including cooperative and sequential binding, cannot fit our data. Notably, lack of coupling between protomers has been well established (Georgieva et al., 2013; Riederer et al., 2018; Erkens et al., 2013; Ruan et al., 2017). Taken together, the data suggest there are two dominant binding states, although there could be additional underlying complexity. Furthermore, it is likely that these two states represent two different conformations of the same site in the transporter population rather than two distinct binding sites within a protomer, since the sums of the apparent stoichiometries (*n*_1_ and *n*_2_ values) averaged 0.97 ± 0.26 (range of 0.74–1.53, *n* = 6; Tables S1 and S2). The bimodal isotherms reflect the presence of a conformation with a higher affinity and lower exothermic binding enthalpy (OFS1) and a conformation with a lower affinity and higher exothermic enthalpy (OFS2; Brautigam, 2015; Le et al., 2013). The two conformations must interconvert only slowly, or not at all, during the ITC experiment to manifest two distinct binding states. We do not observe bimodal isotherms in 50 mM NaCl (Fig. 1 c), likely because the lower substrate affinity at lower Na^+^ concentrations (Reyes et al., 2013; Boudker et al., 2007) blurs the distinctions between the states.

It is possible, in principle, that heterogeneous binding reflects incomplete occupancy of the sodium-binding sites. P-GltPh does not have Na1 due to D405N mutation, and Na2 requires aspartate binding and closure of HP2. Thus, Na3 is the only site that could have incomplete occupancy before substrate binding. However, the measured Na^+^ *K*_*D*_-s for WT Glt_Ph_, 99–170 mM (Riederer and Valiyaveetil, 2019; Reyes et al., 2013; Hanelt et al., 2015), suggests that the sites are saturated at 500 mM NaCl. Furthermore, if the lower-affinity OFS2 were due to incomplete occupancy of the sodium sites, increasing NaCl concentration would eliminate it. Instead, in 1 M NaCl, we observed qualitative differences in the isotherms, consistent with an increased fraction of OFS2 (Fig. 1 e). Thus, the relative populations are unlikely to depend on the occupancy of the Na3 binding site, and NaCl-dependent changes might reflect general salting effects.

To test this, we determined OFS2 fraction in the presence of 500 mM sodium salts containing anions on different ends of the Hofmeister lyotropic series (gluconate^−^ < Cl^−^ < NO_3_^−^; Zhang and Cremer, 2006). Gluconate and nitrate have the opposite effects on protein structure; respectively, they decrease and increase the solubility of nonpolar moieties: “salting out” and “salting in” effects. The biphasic shape of the binding isotherms is less pronounced in gluconate than chloride and nitrate (Fig. 1, d, f, and g). The fitted OFS2 fraction increases from ∼28% in NaCl to ∼38% in NaNO_3_ (Fig. 1 h). It decreases in Na^+^-gluconate, although the binding parameters were difficult to model reproducibly. Decreasing temperature to 10°C resulted in the OFS2 fraction falling to ∼20% in NaCl (Tables S1 and S2). Increasing temperatures above 15°C resulted in protein aggregation.

The observation that temperature and chaotropic salts increase the OFS2 fraction suggests that OFS1 and OFS2 are in a slow equilibrium. Because later ions in the Hofmeister series favor OFS2, the state might feature greater solvent exposure of hydrophobic regions than OFS1. It is possible that looser helical packing leads to more extensive water accessibility, with energy costs offset by increased conformational entropy. We estimated the free energy differences between OFS1 and OFS2 based on the measured populations and binding free energies at 10°C and 15°C under the assumption that the states do not exchange significantly during the ITC experiment (Fig. 1 i). OFS1 and OFS2 are nearly isoenergetic before substrate binding, but OFS1 predominates when bound to L-Asp, reflecting higher affinity. Transiency of OFS2 may explain why alternate substrate-binding conformations have not been visible in structures. Nevertheless, it must be kinetically stable over the course of ITC titrations. Notably, nanomolar apparent binding affinities measured for P-Glt_Ph_ suggest that substrate dissociation contributes little to the binding process observed in ITC. Thus, the two states may differ primarily in the binding on-rates, with the high-affinity state binding substrate faster than the low-affinity state. Regardless, these results strongly suggest that there is conformational heterogeneity in the transporter, manifesting in different binding mechanisms.

### Heterogeneous substrate binding in WT Glt_Ph_

**Figure 2.**
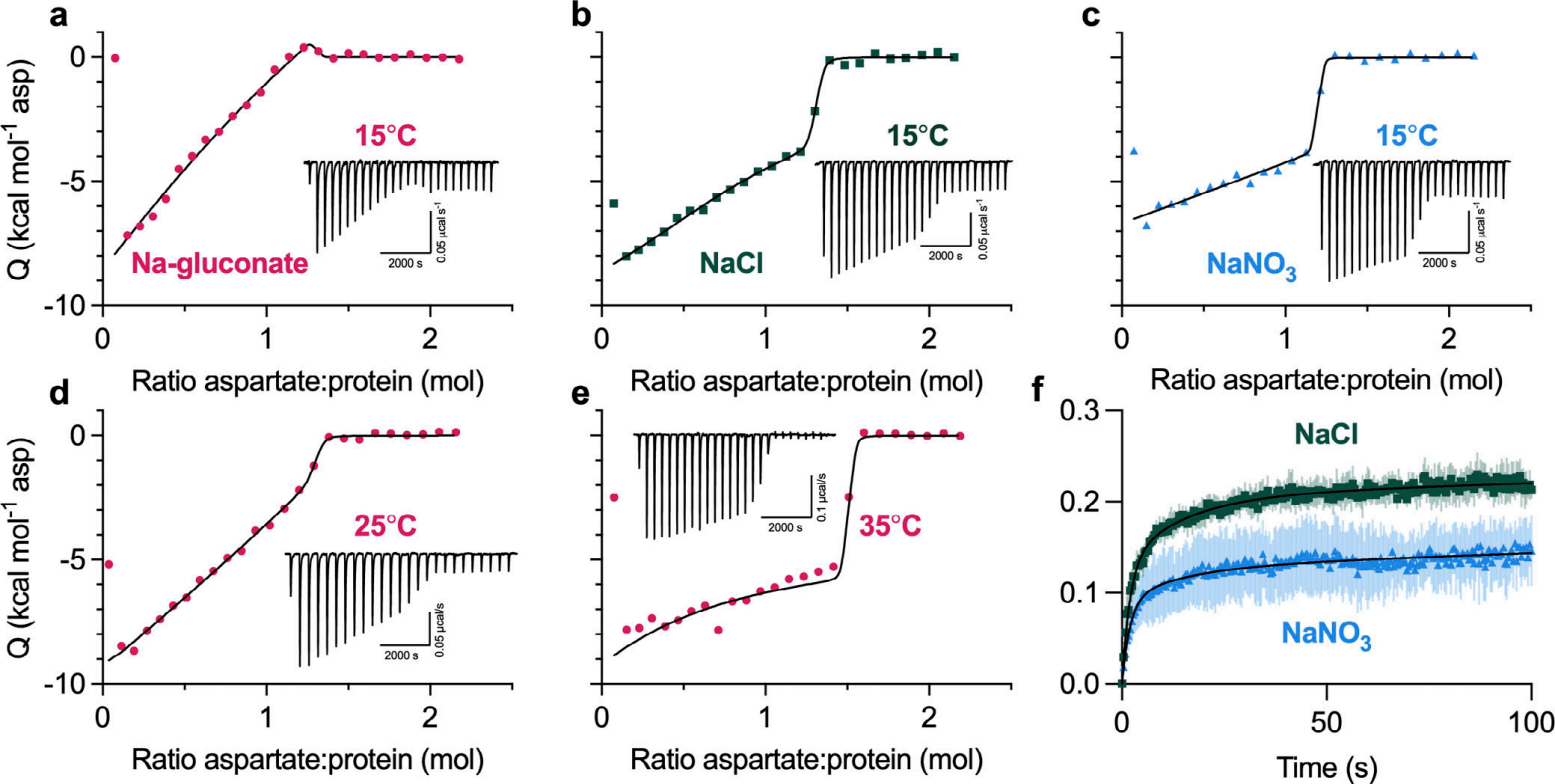
**Heterogeneous substrate binding in WT Glt**_**Ph**_**. (a–c)** Aspartate binding isotherms derived from the ITC experiments performed at 15°C in the presence of 500 mM Na-gluconate (red circles; a); NaCl (green squares; b); or NaNO_3_ (blue triangles; c). **(d and e)** Aspartate binding isotherms in 500 mM Na-gluconate at 25°C (d) or 35°C (e). Experiments in a–e were performed at least twice on independently prepared protein samples, producing similar results. All data were fitted to the two-state model (black lines); however, the binding parameters were not uniquely determined (see Fig. S2 for further information). Insets show the thermal power with the corresponding scales. **(f)** Aspartate transport of Glt_Ph_ (C321A/N378C) was measured using the single-transporter FRET-based assay in NaNO_3_ (blue) or NaCl (green). Transport was initiated by perfusing surface-immobilized proteoliposomes with buffer containing 200 mM sodium salt, 1 μM valinomycin, and 1 μM L-Asp. Data are shown as fractions of total observable transport over time, fitted to triexponential functions (black lines). The fitted parameters are in Fig. S4 b. Data are means and SE from three independent experiments.

We also performed ITC experiments on the WT protein. When we used high protein concentrations to increase experimental sensitivity, we observed unusual features in binding isotherms (Fig. 2, a–c). Specifically, the initial L-Asp injections do not have constant heats as expected for a high-affinity single-site binding process. Instead, injection heats steadily decrease until an abrupt drop occurs when the ligand saturates the protein. The two-state model, where the two affinities are close but not identical and the higher-affinity state has a higher exothermic binding enthalpy, fits data well, though the binding parameters are not uniquely determined (Fig. S2 a). As in P-Glt_Ph_, we observed qualitative differences in the isotherms in different salts and temperatures. The isotherm in 500 mM Na-gluconate at 15°C has a particularly unusual shape (Fig. 2 a) but becomes more reminiscent of a single-site binding in more chaotropic salts (Fig. 2, b and c) or at higher temperatures (Fig. 2, d and e). As in P-Glt_Ph_, changes in the state populations can account for the changing isotherm shapes (Fig. S2 b), though faster exchange between states or altered enthalpies could also contribute. Collectively, our data suggest that WT Glt_Ph_ has multiple binding states in temperature- and salt-modulated equilibrium.

WT transporter in saturating Na^+^ concentrations is predominantly in the OFS (Akyuz et al., 2015; Akyuz et al., 2013), but we cannot exclude that inward-facing protomers contribute to heterogeneous L-Asp binding. However, binding of the transport blocker TFB-TBOA, which has a 100-fold preference for OFS (Mcilwain et al., 2016; Boudker et al., 2007; Wang and Boudker, 2020), also produced bimodal ITC isotherms reminiscent of P-Glt_Ph_ (Fig. S3, a–c). Due to the lower TFB-TBOA affinity, we could not determine precise binding parameters and quantify the salt effects. When Glt_Ph_ saturated with TFB-TBOA was competed with L-Asp, we again observed bimodal isotherms (Fig. S3, d–f). Thus, the inhibitor binding is heterogeneous, and some level of conformational heterogeneity persists after binding.

We used a recently developed smFRET-based single-transporter assay to test if salt-modulated state populations correlated with transport rates (Ciftci et al., 2020). P-Glt_Ph_ C321A/N378C mutant was labeled with PEG_11_-biotin and *N*-ethylmaleimide and reconstituted into liposomes; low protein-to-lipid ratios enriched vesicles containing at most one Glt_Ph_ trimer. The proteoliposomes were then loaded with periplasmic glutamate/aspartate binding protein (PEB1a) Y198F/N73C/K149C mutant with reduced aspartate affinity labeled with maleimide-activated donor (LD555P) and acceptor (LD655) fluorophores (referred to altogether as ccPEB1a-Y198F). The proteoliposomes were immobilized in microscope chambers via biotinylated transporter and assayed for transport after perfusion with saturating Na^+^ and L-Asp concentrations. An increase in mean *E*_*FRET*_ from ∼0.6 to ∼0.8 reflects saturation of the ccPEB1a-Y198F sensor by L-Asp molecules transported into vesicles. This assay previously established kinetic heterogeneity in WT Glt_Ph_ transport (Ciftci et al., 2020), where at least three observable populations (fast, intermediate, and slow) transport at vastly different rates, and all contribute to mean uptake measured in bulk. Most WT transporters are slow, with turnover times of tens to hundreds of seconds.

Because Glt_Ph_ mediates an uncoupled anion conductance, which dissipates the buildup of membrane potential due to electrogenic transport, it shows faster uptake in the presence of more permeant anions (gluconate^−^ < Cl^−^ < NO_3_^−^; Ryan and Mindell, 2007). Thus, we measured transport in K^+^-loaded proteoliposomes in the presence of ionophore valinomycin clamping the potential. Triexponential fits of the uptake kinetics suggest that most molecules are in the slow transporting population regardless of the salt, as expected. The fractions of the slow transporters were similar in Na-gluconate (81.1 ± 3.1) and NaCl (79.5 ± 3.4%) conditions but increased in NaNO_3_ conditions (87.3 ± 2.2%; Fig. 2 f and Fig. S4, a and b). Therefore NaNO_3_-favored OFS2 conformation might correlate with a slower transporter population.

### Transient transport domain structures following substrate binding

smFRET has shown that P-Glt_Ph_ is exclusively outward facing, making this mutant an excellent model to dissect differences between OFS1 and OFS2 using cryo-EM, where structural heterogeneity should reflect the binding heterogeneity. Because OFS2 is transient and OFS1 predominates at equilibrium, we optimized conditions to increase the probability of imaging OFS2. ITC analysis showed that elevated temperatures and chaotropic salts increased the OFS2 fraction (Fig. 1). Thus, we pre-equilibrated P-Glt_Ph_ in 250 mM NaNO_3_ at 25°C and froze grids within ∼5 s after adding 1 mM L-Asp (Data S1).

**Figure 3.**
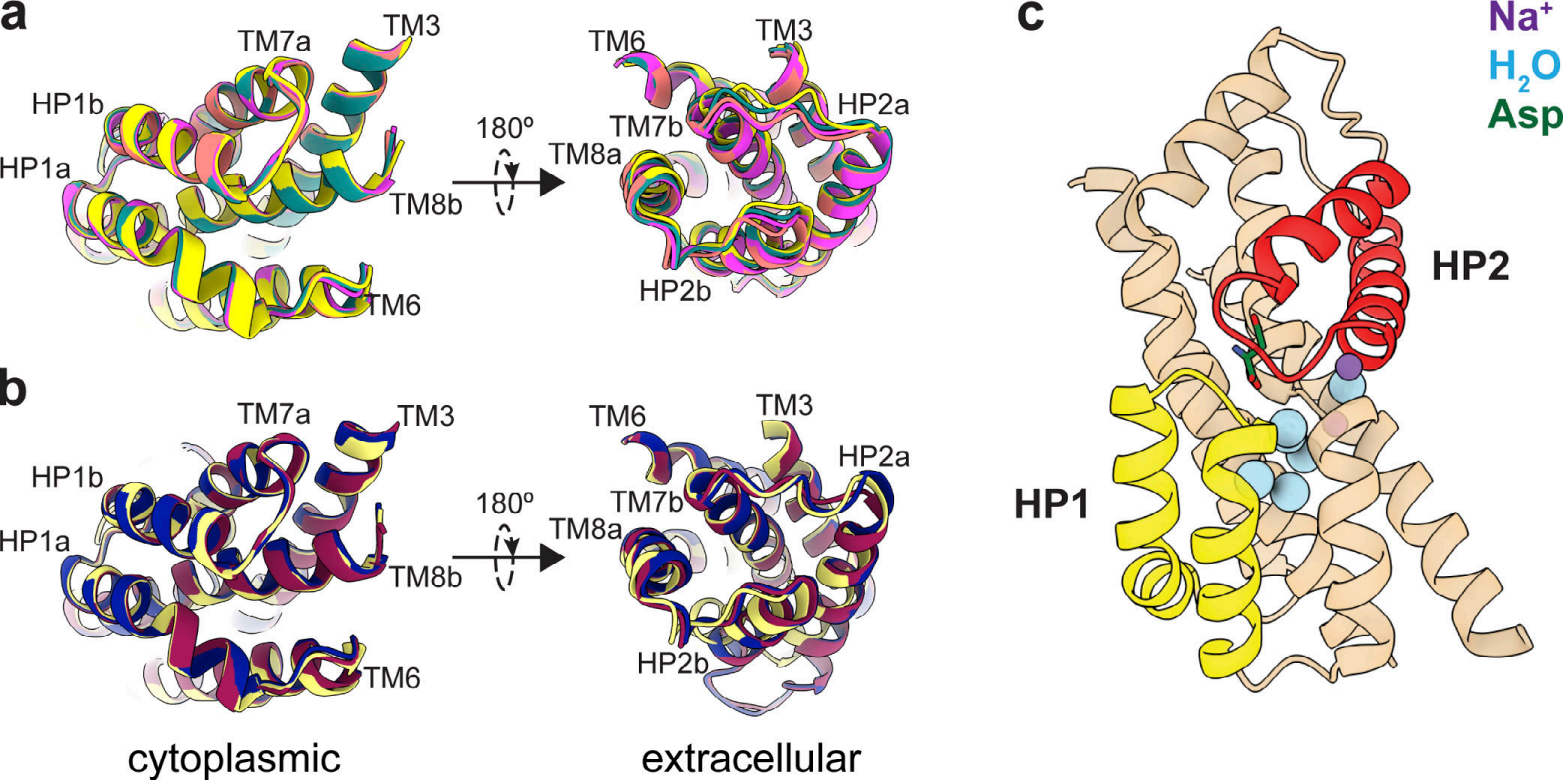
**Mobility of transport domains helices. (a)** Superimposition of transport domains from Data S1. A1 is salmon, A3 is teal, A6 is yellow, and A7 is magenta. **(b)** Superimposition of crystal structures of substrate-bound (PDB accession no. 2NWX; light yellow), TBOA-bound (PDB accession no. 2NWW; dark blue), and Na^+^-bound (PDB accession no. 7AHK; maroon) Glt_Ph_. The domains were superimposed on HP1 and TM7a (residues 258–309). The views are from the intracellular (left) and extracellular (right) sides of the transport domain. **(c)** Cartoon representation of the transport domain resolved in the equilibrated Data S2 after refinement in C3. Resolved waters are shown as blue spheres. Sodium ions are purple spheres, the substrate is green, HP1 is yellow, and HP2 is red.

When we refined particles with imposed C3 symmetry, we obtained density maps with an overall resolution of 3.0 Å (Fig. S5). To maximally retain heterogeneity, we used a data processing approach designed to pick the highest-quality particles regardless of conformation (Su et al., 2020). We then used symmetry expansion and focused classification of single protomers into 10 classes followed by local refinement, a processing approach that previously revealed OFS and iOFS in the WT Glt_Ph_ ensemble (Huang et al., 2020). We did not find any iOFS classes and observed only OFS classes with similar overall structures. We refined models for four classes with the highest resolution, from 3.15 to 3.85 Å (Figs. S5 and S6, and Table S3). When we superimposed their isolated transport domains on the intracellular regions (HP1, TM8b, and TM7a), below the substrate-binding site, they aligned well (Fig. 3 a). In contrast, we observed displacements of helices in the extracellular halves above the substrate-binding site, most noticeable in HP2, TM8a, and TM7b (Fig. 3 a and Video 1). These observations suggest heterogeneity in packing of the extracellular half of the transport domain immediately after substrate binding. Notably, we did not observe any differences between the Na3 sites of these structures, further suggesting that heterogeneous substrate binding does not result from incomplete Na3 occupancy. Similar superpositions of transport domains from the previously reported structures of substrate-, inhibitor-, and Na^+^-only bound Glt_Ph_ (Alleva et al., 2020; Boudker et al., 2007; Yernool et al., 2004) also picture differences in positions of TM7b and TM8a in addition to the expected differences in gating HP2 (Fig. 3 b). These observations further support conformational lability of these helices and suggest that ligand binding entails the restructuring of the entire region.

**Video 1.** Different viewing angles of structures in Fig. 3 a.

### High-resolution equilibrated structures of P-Glt_Ph_

ITC experiments suggest that the low-affinity OFS2 should be transient in the presence of substrate, and the high-affinity OFS1 should predominate at equilibrium (Fig. 1 i). To visualize OFS1, we imaged P-Glt_Ph_ in equilibrium conditions, purified in the presence of 250 mM NaNO_3_ and 1 mM L-Asp (Data S2). We refined the maps to 2.2 Å after imposing C3 symmetry (Fig. S7). The increased resolution compared with Data S1 may reflect reduced structural heterogeneity but can also be due to different microscopes, imaging parameters, or variations in grid preparations. The D405N mutation abolishes Na^+^ binding at Na1 (Boudker et al., 2007; Riederer et al., 2021 *Preprint*); in its place, we observed an excess density, suggesting that a water molecule replaces the ion (Fig S8 a). The S279E side chain points into the extracellular milieu, away from the transport domain (Fig. S8 b).

We found that the equilibrated transport domain structure is nearly identical to class A3 (with overall RMSD of 0.30 Å). Thus, we propose that this conformation corresponds to the higher-affinity OFS1 substate. The ensemble of other conformations observed in Data S1 (A1, A6, and A7), showing different helical packing of the transport domain, together make up the low-affinity OFS2 substate. Previously described V366A and A345V mutations in HP2 are on the interface between the hairpin and TM8a and TM7b helices, where they can disrupt the helical packing. Indeed, HDX-MS measurements suggested increased local dynamics in this region in Y204L/A345V/V366A GltPh mutant. Consistent with our hypothesis, these mutations also decreased substrate affinity, even though the crystal structure of the mutant pictured preserved substrate coordination and binding site details (Huysmans et al., 2021; Ciftci et al., 2021).

Interestingly, we observed several water densities within the transport domain of the equilibrated structure contributing to the hydrogen bond network between substrate- and ion-coordinating residues. All six resolved buried water molecules are below the substrate-binding site. In contrast, the extracellular half of the domain, corresponding to the labile helices in Data S1, appears “dry” (Fig. 3 c). We speculate that the extensive hydrogen bond network in the cytoplasmic half of the transport domain ensures its rigidity. In contrast, the extracellular half, less constrained by polar interactions, can sample multiple conformations with altered packing. Notably, chaotropic salts and elevated temperature favor OFS2 consistent with less well-packed, more dynamic, water-accessible structures.

We also looked for structural heterogeneity in Data S2. 3DVA analysis on a single protomer using the symmetry-expanded particle stack (Punjani and Fleet, 2021) revealed small movements of the transport domain (Videos 2 and 3). Focused 3-D classification of ∼1.6 million symmetry-expanded particles showed only protomers in OFS and yielded structural classes corresponding to the density variations seen in 3DVA analysis. We refined these classes to 2.36–2.65 Å resolutions (Figs. S9 and S10; and Table S4). Superimpositions of the refined trimers on trimerization regions (residues 150–195) showed three subtly different tilts of the classified protomers, consisting of movements of the transport domain and the peripheral parts of the scaffold (OFS_out_, OFS_mid_, and OFS_in_; Video 4). The largest tilt difference of 2.1° is between OFS_out_ and OFS_in_ transport domains. The tilt differences for OFS_out_/OFS_mid_ and OFS_mid_/OFS_in_ were ∼1.1° each (Fig. S11). The adjacent protomers are unaffected, suggesting that the movements occur independently in individual protomers (Video 4 and Fig. S12). Notably, we observed no rearrangements of the extracellular helices regardless of the tilts, and all tilt states most closely resembled class A3 in Data S1, consistent with the high-affinity OFS1 predominating at equilibrium (Fig. S13 and Table S5). The mechanistic basis of the tilts and their role in transport remain unclear. Similar transport domain tilts might also be present in Data S1; however, the moderate resolution of the maps prevents their visualization.

**Video 2.** **3DVA component approximating transitions from OFS**_**out**_ **to OFS**_**mid**_**.**

**Video 3.** **3DVA component approximating transitions from OFS**_**mid**_ **to OFS**_**in**_**.**

**Video 4.** **Structural transitions between OFS**_**out**_ **(pink), OFS**_**mid**_ **(green), and OFS**_**in**_ **(blue).** Models were superimposed on immobile regions of all three protomers (residues 150–195).

**Figure 4.**
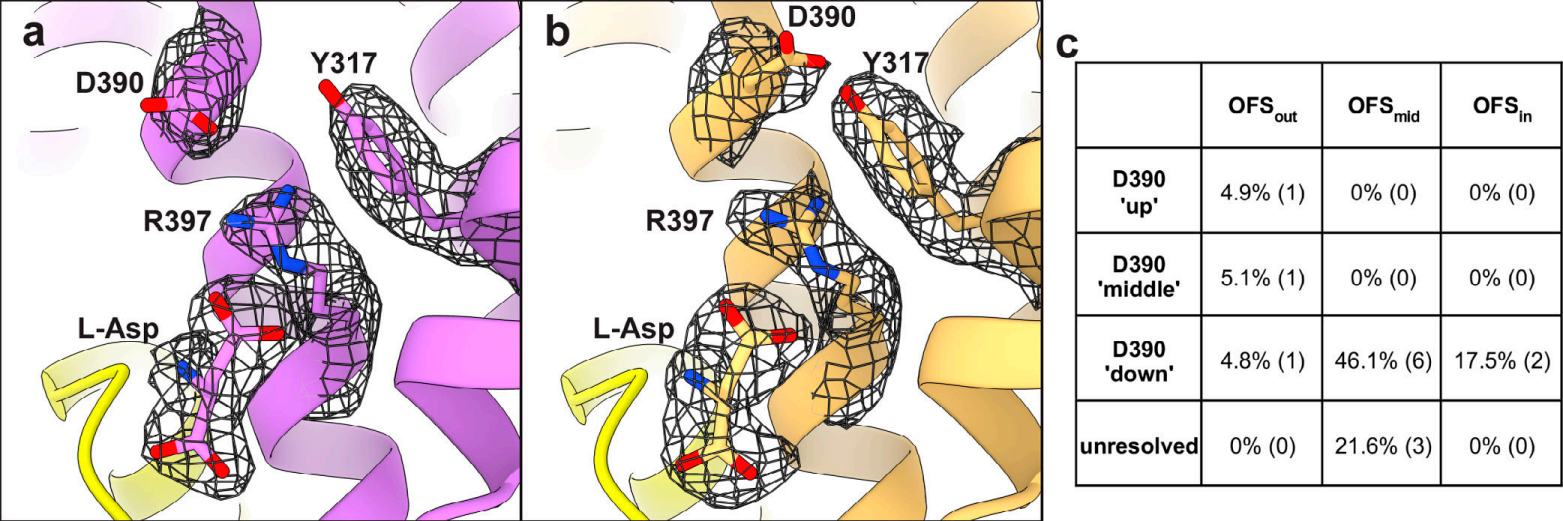
**Multiple rotameric states of D390. (a and b)** Cartoon representations of OFS_out_ classes with down (a) and up (b) D390 rotamers. The mesh objects are density maps contoured at 4 σ; only densities within 1.5 Å of labeled residues are displayed for clarity. HP1 is in yellow, and HP2 was removed for clarity. **(c)** Percentage of particles (total = 1,623,456) that classified into certain D390 rotamers. Numbers in parentheses represent the number of 3-D classes, further detailed in Fig. S9 and Table S6.

Analysis of the substrate-binding sites of Data S2 classes revealed that Asp-390 sampled multiple rotameric states. The highly conserved Asp-390 in TM8 does not coordinate the substrate but is critical for high-affinity binding—D390A mutant has a 1,000-fold lower affinity (Riederer and Valiyaveetil, 2019). Arg-397 in TM8 is the principal substrate-coordinating residue. Its guanidinium group forms hydrogen bonds with the L-Asp sidechain carboxylate and cation-π interactions with Tyr-317 in TM7. We found that Asp-390 can be in down or up rotamers, hydrogen-bonding to Arg-397 or Tyr-317, respectively (Fig. 4, a and b). Classifications also revealed a middle rotamer, perhaps representing an average of the two rotamers or a unique state (Fig. 4 c). Different Asp-390 rotamers do not result in an observable change of Arg-397 conformation but might alter the local electrostatics. Furthermore, tyrosine hydrogen bonding through the OH group potentiates cation-π interactions compared with phenylalanine (Gallivan and Dougherty, 1999), and Y317F mutation leads to a 10-fold loss of L-Asp affinity in Glt_Ph_ (Riederer and Valiyaveetil, 2019). Thus, the up and down rotamers might alter substrate affinity. After additional rounds of sorting, we found that only subpopulations of OFS_out_, comprising ∼10% of all particles, featured up or middle rotamers (Fig. 4 c, Table S6, and Fig. S9). Thus, the preference of Asp-390 to hydrogen-bond with Arg-397 or Tyr-317 might be allosterically coupled to the position of the transport domain. Whether these structural heterogeneities contribute to the elevator dynamic or substrate binding heterogeneities is yet unclear.

## Discussion

Serendipitously, we found that P-Glt_Ph_ mutant has two slowly exchanging outward-facing conformational substates, OFS1 and 2, binding aspartate with different affinities and enthalpies (Fig. 1). smFRET and cryo-EM showed that P-Glt_Ph_ is predominantly outward facing (Fig. 1, a and b). Thus, P-Glt_Ph_ is an excellent model to consider the mechanism of heterogeneous binding in OFS. WT Glt_Ph_ is less suitable because its conformational ensemble includes iOFS and IFS (Wang and Boudker, 2020; Reyes et al., 2013; Akyuz et al., 2013). Nevertheless, WT binding isotherms also suggest multiple binding conformations modulated by salts and temperature (Fig. 2). Interestingly, a recent saturation transfer difference NMR study reported an unusually low Hill coefficient of 0.69 for aspartate binding to liposome-reconstituted Glt_Ph_ (Hall et al., 2020). A Hill coefficient below one may reflect negative cooperativity or result from multiple binding states with distinct affinities (Cattoni et al., 2015; Sevlever et al., 2020; Wang and Pan, 1996). Distinguishing these possibilities requires a kinetic approach (Cattoni et al., 2009).

Recent studies showed that Glt_Ph_, originating from a hyperthermophilic archaeon, exhibits activity modes at ambient temperature, where transporter subpopulations function with rates differing by orders of magnitude (Ciftci et al., 2020). Switching between the modes is rare, occurring on a timescale of hundreds of seconds. These modes were attributed to subpopulations with different elevator dynamics and intracellular substrate-release rates (Matin et al., 2020; Huysmans et al., 2021; Ciftci et al., 2021). Here, we observed that NaNO_3_ modulated the populations of the binding substates and increased the fraction of the slow transporters (Fig. 2 f). Therefore, the substrate-binding heterogeneity might contribute to the heterogeneous uptake rates or reflect different substrate-binding properties of fast and slow subpopulations. Some gain-of-function mutations, including R276S/M395R, A345V, and V366A, both increase Glt_Ph_ elevator dynamics and reduce substrate affinity (Huysmans et al., 2021; Ciftci et al., 2021). Thus, structural perturbations can affect binding and translocation in concert, suggesting that the two processes share some of the determinants. Therefore, insights into the structural bases of heterogeneous binding might also illuminate the origins of heterogeneous transport kinetics.

Our cryo-EM structures of P-Glt_Ph_, imaged in conditions nearly identical to the ITC experiments, demonstrate that the extracellular half of the transport domain has a continuum of packing states upon binding (Fig. 3, a and b). Yet, after equilibration, we observed no such heterogeneity. We found no other pronounced structural heterogeneities that exist immediately after binding but are gone after equilibration. Furthermore, our structural classes do not display any evidence of altered substrate coordination that could explain affinity differences. Thus, heterogeneous binding observed in ITC correlates with different helical packing observed in cryo-EM structures. We propose that structural flexibility in the extracellular regions, including the gating HP2 and adjacent helices, alter the energetics of substrate binding. We further suggest that the structural class with the optimal packing corresponds to the high-affinity substate, and the classes with the continuum of helical packing arrangements together correspond to the low-affinity substate. In this model, following substrate binding, the transporter relaxes to the optimally packed state on a very slow time scale. The equilibration process involves helical repacking and might be slow because it requires rearrangement of a large transport domain or substrate release and rebinding. Consistently with our hypothesis, packing mutations A345V and V366A in the extracellular helices lead to decreased affinity and increased binding and dissociation kinetics (Huysmans et al., 2021; Ciftci et al., 2021). Notably, the closure of the HP2 tip over the substrate-binding site is unlikely to contribute to binding heterogeneity because it is a rapid process (Riederer and Valiyaveetil, 2019), and achieving tight binding requires a kinetically slow step (Ewers et al., 2013; Hanelt et al., 2015). Thus, we see helical repacking as the most, if not the only, feasible explanation of the experimentally observed heterogeneous binding.

Our structural analysis suggests that there is a structural specialization within the transport domain of glutamate transporters. A network of buried waters and polar residues within the cytoplasmic half of the domain (TM8b, TM7a, and HP1) might ensure exquisitely specific, evolutionarily conserved structure responsible for sodium selectivity and allosteric coupling between ion and substrate binding (Fig. 3 c). In contrast, the extracellular half (TN7b, HP2, and TM8a) is more hydrophobic and contains no resolved waters. Fewer constraining polar interactions may permit variable helical packing in this region, setting the dynamic properties—substrate affinities and elevator dynamics—of Glt_Ph_ substates and, perhaps, different glutamate transporter homologues. Consistently, extensive mutagenesis in HP1 and adjacent helices did not identify gain-of-function mutants analogous to those in HP2 (Huysmans et al., 2021). These observations of structural specializations are reminiscent of “protein sectors,” where proteins can have discrete, independently evolving functional units in tertiary structure (Halabi et al., 2009).

The structural differences between the high- and low-affinity states are small and require analysis of high-resolution cryo-EM datasets. Similarly, modal gating in KcsA was attributed to subtle sidechain rearrangements (Chakrapani et al., 2011). Functional heterogeneity has been observed in ion channels, transporters, and enzymes. For example, nicotinic acetylcholine receptors feature opening bursts interspersed with short or long closed periods (Colquhoun and Sakmann, 1985), and ionotropic glutamate receptors show complex kinetics with multiple gating modes (Popescu, 2012). P-type ATPase also displayed periods of rapid transport interspersed with prolonged pauses (Veshaguri et al., 2016). As in Glt_Ph_, these distinct modes may be due to concerted subtle restructuring of protein regions occurring on long timescales.

## Supplementary Material

Table S1 shows L-Asp binding to P-Glt_Ph_ (S279E/D405N) at 10°C in 500 mM NaCl.

Table S2 shows L-Asp binding to P-Glt_Ph_ (S279E/D405N) at 15°C in 500 mM NaCl.

Table S3 shows model refinement and validation statistics for Data S1.

Table S4 shows model refinement and validation statistics for Data S2.

Table S5 shows comparison between structures from Data S1 and tilt states from Data S2.

Table S6 shows correlation of tilt states and D390 rotamers from Data S2 processing.

Data S1 shows maps and models generated from P-Glt_Ph_ purified in substrate-free (Na^+^ only) conditions, and substrate (L-Asp) was added ~5 s prior to freezing.

Data S2 provides data maps and models generated from P-Glt_Ph_ purified in the presence of substrate and ions (Na^+^ and L-Asp).
