# Supplementary material for: The archaeal glutamate transporter homologue GltPh shows heterogeneous substrate binding
Source: J Gen Physiol. 2022 Apr 22;154(5):e202213131. doi: 10.1085/jgp.202213131 (PMC9044058; doi:10.1085/jgp.202213131)
Supplement: Table S1 — shows L-Asp binding to P-GltPh (S279E/D405N) at 10°C in 500 mM NaCl. [file JGP_202213131_TableS1.docx]

| ***Trial*** | ***n_1_*** | ***K_D,1_ (M)*** | ***ΔG_1_***  ***(kcal mol^-1^)*** | ***ΔH_1_***  ***(kcal mol^-1^)*** | ***ΔS_1_***  ***(cal mol^-1^ K^-1^)*** | ***n_2_*** | ***K_D,2_ (M)*** | ***ΔG_2_***  ***(kcal mol^-1^)*** | ***ΔH_2_***  ***(kcal mol^-1^)*** | ***ΔS_2_***  ***(cal mol^-1^ K^-1^)*** | ***% n_2_*** |
| --- | --- | --- | --- | --- | --- | --- | --- | --- | --- | --- | --- |
| **1** | 0.61 | 2.3e-9 | -11.35 | -2.06 | 32.2 | 0.13 | 1.3e-7 | -8.98 | -6.00 | 10.3 | 18 |
| **2** | 0.64 | 1.0e-10 | -13.15 | -1.64 | 40.0 | 0.20 | 5.5e-9 | -10.81 | -4.73 | 21.1 | 24 |
| **3** | 0.78 | 3.6e-9 | -11.10 | -2.13 | 31.1 | 0.18 | 1.1e-7 | -9.07 | -6.35 | 9.4 | 19 |
| **avg** |  |  | **-11.86**  **± 0.91** | **-1.94**  **± 0.22** | **34.4**  **± 3.9** |  |  | **-9.62**  **± 0.84** | **-5.70**  **± 0.69** | **13.6**  **± 5.3** | **20**  **± 3** |

**Supplementary Table 1. L-Asp binding to P-Glt_Ph_ (S279E/D405N) at 10°C in 500 mM NaCl.** Binding parameters are from fits to the two-state model. Averaged values are means and standard deviations from three independent experiments.
