# Supplementary material for: The archaeal glutamate transporter homologue GltPh shows heterogeneous substrate binding
Source: J Gen Physiol. 2022 Apr 22;154(5):e202213131. doi: 10.1085/jgp.202213131 (PMC9044058; doi:10.1085/jgp.202213131)
Supplement: Table S2 — shows L-Asp binding to P-GltPh (S279E/D405N) at 15°C in 500 mM NaCl. [file JGP_202213131_TableS2.docx]

| ***Trial*** | ***n_1_*** | ***K_D,1_ (M)*** | ***ΔG_1_***  ***(kcal mol^-1^)*** | ***ΔH_1_***  ***(kcal mol^-1^)*** | ***ΔS_1_***  ***(cal mol^-1^ K^-1^)*** | ***n_2_*** | ***K_D,2_ (M)*** | ***ΔG_2_***  ***(kcal mol^-1^)*** | ***ΔH_2_***  ***(kcal mol^-1^)*** | ***ΔS_2_***  ***(cal mol^-1^ K^-1^)*** | ***% n_2_*** |
| --- | --- | --- | --- | --- | --- | --- | --- | --- | --- | --- | --- |
| **1** | 1.13 | 1.9e-9 | -11.51 | -3.05 | 29.4 | 0.39 | 3.9e-8 | -9.77 | -6.63 | 10.9 | 26 |
| **2** | 0.64 | 1.3e-9 | -11.73 | -3.31 | 29.2 | 0.22 | 6.1e-8 | -9.51 | -7.13 | 8.3 | 26 |
| **3** | 0.63 | 3.4e-9 | -11.17 | -2.11 | 31.4 | 0.29 | 1.7e-8 | -10.23 | -9.04 | 4.2 | 31 |
| **avg** |  |  | **-11.47**  **± 0.23** | **-2.82**  **± 0.52** | **30.0**  **± 1.0** |  |  | **-9.84**  **± 0.30** | **-7.60**  **± 1.04** | **7.8**  **± 2.8** | **28**  **± 3** |

**Supplementary Table 2. L-Asp binding to P-Glt_Ph_ (S279E/D405N) at 15°C in 500 mM NaCl.** Binding parameters were fitted to the two-state model. Averaged values are means and standard deviations from three independent experiments.
