# Supplementary material for: The archaeal glutamate transporter homologue GltPh shows heterogeneous substrate binding
Source: J Gen Physiol. 2022 Apr 22;154(5):e202213131. doi: 10.1085/jgp.202213131 (PMC9044058; doi:10.1085/jgp.202213131)
Supplement: Table S3 — shows model refinement and validation statistics for Data S1. [file JGP_202213131_TableS3.docx]

|  | P-Glt_Ph_ A1 | P-Glt_Ph_ A3 | P-Glt_Ph_ A6 | P-Glt_Ph_ A7 |
| --- | --- | --- | --- | --- |
| **Cryo-EM acquisition and processing** | | | | |
| EMDataBank ID | EMD-24406 | EMD-24406 | EMD-24406 | EMD-24406 |
| Symmetry imposed | C1 | C1 | C1 | C1 |
| Initial particles | 1,174,359 | 1,174,359 | 1,174,359 | 1,174,359 |
| Class number | A1 | A3 | A6 | A7 |
| Final particles (protomers) | 102,463 | 142,081 | 81,886 | 99,100 |
| Resolution (masked FSC = 0.143, Å) | 3.42 | 3.06 | 3.65 | 3.53 |
| Density modified CC (0.5, Å) | 3.45 | 3.09 | 3.64 | 3.57 |
| **Model Refinement** | | | | |
| Model resolution (FSC = 0.50/0.143 Å) | 3.55 / 3.08 | 3.15 / 2.63 | 3.85 / 3.35 | 3.76 / 3.32 |
| **Model composition** |  |  |  |  |
| Non-hydrogen atoms | 3,097 | 3,094 | 3,094 | 3,094 |
| Protein residues | 416 | 416 | 416 | 416 |
| Ligands | 2 | 2 | 2 | 2 |
| **R.m.s. deviations** |  |  |  |  |
| Bond lengths (Å) | 0.003 | 0.002 | 0.003 | 0.003 |
| Bond angles (°) | 0.486 | 0.462 | 0.578 | 0.523 |
| **Validation** |  |  |  |  |
| MolProbity score | 1.42 | 1.21 | 1.58 | 1.78 |
| Clash score | 6.93 | 4.41 | 10.55 | 10.55 |
| Poor rotamers (%) | 0 | 0 | 0 | 0 |
| **Ramachandran plot** |  |  |  |  |
| Favored (%) | 97.82 | 98.79 | 97.82 | 96.37 |
| Allowed (%) | 2.18 | 1.21 | 2.18 | 3.63 |
| Disallowed (%) | 0 | 0 | 0 | 0 |

**Supplementary Table 3.** Model refinement and validation statistics for Data S1.
