# Supplementary material for: The archaeal glutamate transporter homologue GltPh shows heterogeneous substrate binding
Source: J Gen Physiol. 2022 Apr 22;154(5):e202213131. doi: 10.1085/jgp.202213131 (PMC9044058; doi:10.1085/jgp.202213131)
Supplement: Table S4 — shows model refinement and validation statistics for Data S2. [file JGP_202213131_TableS4.docx]

|  | P-Glt_Ph_ OFS | P-Glt_Ph_ OFS_out_ | P-Glt_Ph_ OFS_out_ | P-Glt_Ph_ OFS_mid_ | P-Glt_Ph_ OFS_in_ |
| --- | --- | --- | --- | --- | --- |
| D390 state |  | down | up | down | down |
| **Cryo-EM acquisition and processing** | | | | | |
| EMDataBank ID | EMD-24405 | EMD-24405 | EMD-24405 | EMD-24405 | EMD-24405 |
| Symmetry imposed | C3 | C1 | C1 | C1 | C1 |
| Particle classification | trimers | protomers | protomers | protomers | protomers |
| Initial particles | 9,234,148 | 1,620,456 | 1,620,456 | 1,620,456 | 1,620,456 |
| Final particles | 503,427 | 78,248 | 80,207 | 243,208 | 142,738 |
| Resolution (masked FSC = 0.143, Å) | 2.20 | 2.56 | 2.61 | 2.37 | 2.40 |
| Density modified CC (0.5, Å) | 2.20 | 2.56 | 2.59 | 2.33 | 2.39 |
| **Model Refinement** | | | | | |
| PDB ID | 7RCP |  |  |  |  |
| Model resolution (FSC = 0.50/0.143 Å) | 2.23 / 2.01 | 2.60 / 2.08 | 2.66 / 2.04 | 2.36 / 1.92 | 2.42 / 2.00 |
| **Model composition** |  |  |  |  |  |
| Non-hydrogen atoms | 9,339 | 9,294 | 9,294 | 9,306 | 9,307 |
| Protein residues | 1,248 | 1,246 | 1,246 | 1,246 | 1,245 |
| Ligands | 6 | 6 | 6 | 6 | 6 |
| Waters | 57 | 29 | 31 | 41 | 49 |
| **R.m.s. deviations** |  |  |  |  |  |
| Bond lengths (Å) | 0.005 | 0.003 | 0.003 | 0.004 | 0.003 |
| Bond angles (°) | 0.620 | 0.494 | 0.488 | 0.618 | 0.550 |
| **Validation** |  |  |  |  |  |
| MolProbity score | 1.18 | 1.26 | 1.38 | 1.07 | 1.15 |
| Clash score | 3.88 | 5.00 | 5.52 | 2.79 | 3.58 |
| Poor rotamers (%) | 0 | 0 | 0 | 0 | 0 |
| **Ramachandran plot** |  |  |  |  |  |
| Favored (%) | 98.79 | 98.54 | 98.62 | 98.62 | 98.78 |
| Allowed (%) | 1.21 | 1.26 | 1.38 | 1.38 | 1.22 |
| Disallowed (%) | 0 | 0 | 0 | 0 | 0 |

**Supplementary Table 4.** Model refinement and validation statistics for Data S2.
