# Supplementary material for: The archaeal glutamate transporter homologue GltPh shows heterogeneous substrate binding
Source: J Gen Physiol. 2022 Apr 22;154(5):e202213131. doi: 10.1085/jgp.202213131 (PMC9044058; doi:10.1085/jgp.202213131)
Supplement: Table S5 — shows comparison between structures from Data S1 and tilt states from Data S2. [file JGP_202213131_TableS5.docx]

| **Class** | **OFS_out_** | **OFS_mid_** | **OFS_in_** | **A1** | **A3** | **A6** | **A7** |
| --- | --- | --- | --- | --- | --- | --- | --- |
| **OFS_out_** |  | 0.201 | 0.234 | 0.457 | 0.331 | 0.571 | 0.511 |
| **OFS_mid_** | 0.201 |  | 0.151 | 0.434 | 0.293 | 0.575 | 0.485 |
| **OFS_in_** | 0.234 | 0.151 |  | 0.429 | 0.301 | 0.577 | 0.499 |
| **A1** | 0.457 | 0.434 | 0.429 |  | 0.389 | 0.565 | 0.506 |
| **A3** | **0.331** | **0.293** | **0.301** | 0.389 |  | 0.500 | 0.448 |
| **A6** | 0.571 | 0.575 | 0.577 | 0.565 | 0.500 |  | 0.536 |
| **A7** | 0.511 | 0.485 | 0.499 | 0.506 | 0.448 | 0.536 |  |

**Supplementary Table 5.** Comparison between structures from Data S1 and tilt states from Data S2. Cα RMSDs were determined using ‘matchmaker’ implemented in ChimeraX using default parameters. Residues 12-106, 130-416 were used for structural alignment to include only well-structured, well-resolved regions. Lowest RMSD values between classes from Data S1 and Data S2 are red/bolded.
