# Supplementary material for: The archaeal glutamate transporter homologue GltPh shows heterogeneous substrate binding
Source: J Gen Physiol. 2022 Apr 22;154(5):e202213131. doi: 10.1085/jgp.202213131 (PMC9044058; doi:10.1085/jgp.202213131)
Supplement: Table S6 — shows correlation of tilt states and D390 rotamers from Data S2 processing. [file JGP_202213131_TableS6.docx]

| **Class**  **(Round 1)** | **Tilt state** | **D390 rotamer** | **Class**  **(Round 2)** | **Tilt state** | **D390 rotamer** |
| --- | --- | --- | --- | --- | --- |
| B1-1 | OFS_out_ | intermediate | B2-1 | OFS_out_ | down |
| B1-2 | OFS_mid_ | unresolved | B2-2 | OFS_mid_ | unresolved |
| B1-3 | OFS_mid_ | down | B2-3 | OFS_mid_ | down |
| B1-4 | OFS_in_ | down | B2-4 | OFS_out_ | up |
| **B1-5** | **OFS_out_** | **ambiguous** | B2-5 | OFS_mid_ | unresolved |
| B1-6 | OFS_mid_ | down | B2-6 | OFS_mid_ | down |
| **B1-7** | **OFS_mid_** | **ambiguous** | B2-7 | OFS_in_ | down |
| B1-8 | OFS_mid_ | down | B2-8 | OFS_mid_ | down |

**Supplementary Table 6. Correlation of tilt states and D390 rotamers from Data S2 processing.**
